# Unravelling functional neurology: does spinal manipulation have an effect on the brain? - a systematic literature review

**DOI:** 10.1186/s12998-019-0265-8

**Published:** 2019-10-02

**Authors:** Anne-Laure Meyer, Michel-Ange Amorim, Martin Schubert, Petra Schweinhardt, Charlotte Leboeuf-Yde

**Affiliations:** 1 CIAMS, Univ. Paris-Sud, Université Paris-Saclay, 91405 Orsay Cedex, France; 2 CIAMS, Université d’Orléans, 45067 Orléans, France; 3 Institut Franco Européen de Chiropraxie, 24 Bd Paul Vaillant Couturier, 94200 Ivry sur Seine, France; 4 Spinal Cord Injury Center, University Hospital Balgrist, Zürich, Switzerland; 5 Integrative Spinal Research Group, Department of Chiropractic Medicine, University Hospital Balgrist and University of Zürich, Zürich, Switzerland; 6 Institute for Regional Health Research, University of Southern Denmark, Odense, Denmark

**Keywords:** Spinal manipulation, Brain, Functional Neurology, Chiropractic, Systematic review, Manipulation vertébrale, Cerveau, Neurologie Fonctionnelle, Chiropraxie, Revue systématique

## Abstract

**Background:**

A recent hypothesis purports that spinal manipulation may cause changes at a brain level. Functional Neurology, a mainly chiropractic approach, promotes the use of spinal manipulation to improve ‘brain function’ as if it were a proven construct. No systematic review has been performed to investigate how well founded this hypothesis is.

**Objective:**

To investigate whether spinal manipulation has an effect on ‘brain function’ that is associated with any clinical benefits.

**Method:**

In this systematic review, the literature was searched in PubMed, Embase, and PEDro (final search February 2018). We included randomized or non-randomized controlled studies, in which spinal manipulation was performed to any region of the spine, applied on either symptomatic or asymptomatic humans, and compared to a sham or to another type of control. The outcome measures had to be stated as direct or proxy markers of ‘brain function’. Articles were reviewed blindly by at least two reviewers, using a quality checklist designed for the specific needs of the review. Studies were classified as of ‘acceptable’, ‘medium’, or ‘low’ methodological quality. Results were reported in relation to (i) control intervention (sham, ‘inactive control’, or ‘another physical stimulus’) and (ii) study subjects (healthy, symptomatic, or with spinal pain” subjects/spinal pain”), taking into account the quality. Only results obtained from between-group or between-intervention comparisons were considered in the final analysis.

**Results:**

Eighteen of 1514 articles were included. Studies were generally of ‘low’ or ‘medium’ methodological quality, most comparing spinal manipulation to a control other than a sham. Thirteen out of the 18 studies could be included in the final analysis. Transitory effects of different types of ‘brain function’ were reported in the three studies comparing spinal manipulation to sham (but of uncertain credibility), in “subclinical neck/spinal pain” subjects or in symptomatic subjects. None of these three studies, of ‘medium’ or ‘acceptable’ quality, investigated whether the neurophysiological effects reported were associated with clinical benefits. The remaining 10 studies, generally of ‘low’ or ‘medium’ quality, compared spinal manipulation to ‘inactive control’ or ‘another physical stimulus’ and similarly reported significant between-group differences but inconsistently.

**Conclusion:**

The available evidence suggests that changes occur in ‘brain function’ in response to spinal manipulation but are inconsistent across and - sometimes - within studies. The clinical relevance of these changes is unknown. It is therefore premature to promote the use of spinal manipulation as a treatment to improve ‘brain function’.

**Electronic supplementary material:**

The online version of this article (10.1186/s12998-019-0265-8) contains supplementary material, which is available to authorized users.

## Résumé

**Introduction:**

Une hypothèse récente propose que la manipulation vertébrale causerait des changements neurophysiologiques au niveau du cerveau. En Neurologie Fonctionnelle, approche principalement présente en chiropraxie, l’utilisation de la manipulation vertébrale est déjà promue comme capable d’améliorer le fonctionnement du cerveau. A ce jour, aucune revue systématique de la littérature n’a été conduite afin de connaître l’étendue et la qualité de l’évidence scientifique susceptible de justifier cet usage de la manipulation vertébrale.

**Objectif:**

Déterminer, à travers une revue systématique et critique de la littérature, si la manipulation vertébrale a un effet (spécifique) sur le cerveau et, si oui, si celui-ci est associé à un effet clinique.

**Méthode:**

Le moteur de recherche PubMed et deux bases de données, Embase et PEDro, ont fait l’objet d’une recherche bibliographique (actualisée en février 2018). Les critères d’inclusion étaient: essais contrôlés, randomisés ou non, dans lesquels la manipulation vertébrale avait été comparée à un placébo ou à un autre type de contrôle, chez des sujets symptomatiques ou asymptomatiques. La manipulation vertébrale pouvait avoir été effectuée au niveau de n’importe quelle région de la colonne vertébrale et les critères de jugement utilisés devaient être indiqués comme capables de mesurer, directement ou indirectement, une forme ‘d’activité cérébrale’. La qualité méthodologique des études a été évaluée de manière indépendante par au moins deux chercheurs à l’aide d’une grille de qualité créée pour les besoins de cette revue. Les études ont été classifiées comme étant de qualité méthodologique ‘acceptable’, ‘moyenne’, ou ‘faible’. Les résultats ont été rapportés de façon narrative, en fonction du type de contrôle utilisé (placébo, ‘inactif’, ou ‘autre stimulus physique’) et du type de sujets d’étude (sans problème de santé, symptomatiques, ou présentant des « douleurs rachidiennes subcliniques »), en tenant compte de la qualité méthodologique. Seuls les résultats issus de comparaisons inter-groupes ont été pris en compte dans notre analyse finale.

**Résultats:**

Dix-huit articles parmi les 1514 titres obtenus ont été inclus. Les études étaient pour la plupart de qualité méthodologique ‘faible’ ou ‘moyenne’ et avaient principalement comparé la manipulation vertébrale à une intervention autre que placébo. Les résultats rapportés dans 13 des 18 articles inclus ont finalement été pris en compte. Un effet transitoire sur différentes formes ‘d’activité cérébrale’ a été rapporté à l’issue de trois études dans lesquelles la manipulation vertébrale avait été comparée à un placébo (de crédibilité cependant incertaine), chez des sujets présentant des « douleurs rachidiennes subcliniques » (*n* = 2) ou souffrant de cervicalgies non spécifiques aiguës / subaiguës (*n* = 1). Une potentielle association avec un effet clinique n’a pas été étudiée dans ces trois études, de qualité méthodologique ‘moyenne’ (*n* = 2) ou ‘acceptable’ (*n* = 1). Dans les 10 études restantes, la plupart de qualité méthodologique ‘faible’ ou ‘moyenne’, la manipulation vertébrale avait été comparée à un contrôle ‘inactif’ ou à un ‘autre stimulus physique’. Des différences inter-groupes y ont également été rapportées, parfois de façon inconsistante.

**Conclusion:**

La littérature scientifique suggère que des changements neurophysiologiques surviennent au niveau du cerveau en réponse à la manipulation vertébrale mais, de façon inconsistante. La pertinence clinique de ces changements n’est pas connue. Ainsi, il est prématuré d’attribuer à la manipulation vertébrale des bénéfices cliniques via un effet sur le cerveau.

## Introduction

Spinal manipulation (SM) is widely used by various health practitioners, including physiotherapists, osteopaths and chiropractors, to treat mainly musculoskeletal conditions, but some also use it for a variety of other health-related problems [1, 2]. While the literature tends to support the benefit of SM as a useful treatment in the musculoskeletal area, no clear evidence exists in relation to non-musculoskeletal conditions [3].

This lack of evidence contrasts with claims of some therapists, including (but not restricted to) those who practice using the theoretical concepts of Functional Neurology (FN), a mainly chiropractic approach, founded by a chiropractor, FR Carrick [4]. In addition to musculoskeletal conditions, “functional neurologists” (i.e. FN practitioners) also provide treatment for complex disorders such as neurodevelopmental disorders, neurodegenerative disorders, and post-traumatic stress disorders [5]. Also based on these FN concepts, some therapists claim to enhance human performances (e.g. physical performances), including in asymptomatic individuals [5].

In line with FN, a current hypothesis is that the clinical benefits observed following SM would be, at least partially, due to neurophysiological changes within the brain [6, 7]. Some practitioners already use this concept claiming it to be a fact that SM has a clinically relevant effect on the brain, as shown through several sources of information in a recent scoping review on FN [5]. Furthermore, for some proponents of this hypothesis, at least for the “functional neurologists”, a multitude of conditions results from dysfunction within the brain [5]. It is stated that malfunctioning clusters of neurons, described as primarily located within the brain, could be the single cause of virtually any type of symptom and/or disorder that a person may have. Examples extracted form a FN textbook are attention deficit and hyperactivity disorder, depression, mechanical low back pain, and migraines [7]. In addition, these ‘malfunctions’ are stated to be reversible through the use of stimulation of the nervous system, including by SM [5]. This would give SM the potential to be used for both musculoskeletal and non-musculoskeletal conditions. In fact, it is even stated that SM is one of the most easily available methods for manual practitioners to improve ‘brain function’ [7].

Within the scientific literature, differently framed hypotheses exist in relation to the potential mechanisms involving the brain, which could explain clinical benefits following SM [6, 8, 9]. The one mechanism that seems to prevail relates to the chiropractic concept of ‘subluxation’, which has developed over time [10]. Currently some authors purport that ‘subluxations’ modify afferent inputs to the central nervous system [6, 11]. These authors state, in addition to this, that the ‘subluxation’ is at the source of maladaptive neural plastic changes, including in the cerebral cortex, which in turn result in altered processing and integration of subsequent afferent inputs and, consequently, altered motor outputs [11]. As a consequence, SM is claimed to restore afferent inputs to the central nervous system (including to the brain) and result in appropriate motor outputs from the central nervous system [11].

Potential neurophysiological effects of SM on the brain have been the focus of several recent experimental studies. As the brain is involved in a multitude of functions, its activities or alteration of activities after an intervention can be explored in several ways. Not surprisingly then, the studies in this field of research use various approaches and outcome measures to test the hypothesis that SM has an effect on ‘brain function’. For example, some studies investigated the potential effect of SM on brain areas involved in pain processing [9] and autonomic functions [8], whereas others reported on the potential effect of SM on cortical somatosensory integration of stimuli from the upper limb [6]. Therefore, in the present systematic review ‘brain function’ is used as a generic expression referring to processes in which the brain is involved.

Because studies are quite heterogeneous, it is difficult to understand and interpret the evidence in this area. Nevertheless, this task is needed to understand if assertions of the ‘brain-mediated’ hypothesis proponents are substantiated by scientific evidence. A narrative review on the topic by Haavik and Murphy was published in 2012 [6]. In this review, they concluded that some evidence supports a brain mechanism of action for SM but that it remains to be investigated whether this correlates to clinical benefits. They also stated that such studies were underway [6]. Given that 7 years have passed since this narrative review, the aforementioned studies exploring potential associations between clinical and ‘brain function’ changes post-SM were likely published, and thus may provide important updates to the state of the field. For these reasons, we undertook a systematic critical review of the literature, which had as its overall aim to investigate whether SM has an effect on ‘brain function’ that is associated with any clinical benefits in healthy and/or symptomatic subjects. The specific research questions were:

- **In relation to sham controlled studies, i.e. ‘** ***effect’*** **studies:**
  1. Is there an effect of SM on ‘brain function’?
  2. If there is an effect, for how long does it last?
  3. If there is an effect, is it associated with any clinical benefits?
- **In relation to other controlled studies (‘inactive control’ or ‘another physical stimulus’), i.e. the ‘** ***differences in outcome’*** **studies:**
  4 - Is there a difference in ‘brain function’ after SM vs. an ‘inactive control’?
  5 - Is there a difference in ‘brain function’ after SM vs. ‘another physical stimulus’?

## Methods

A systematic critical review of the literature was carried out to shed light on the research questions above. The review was registered in the PROSPERO international prospective register of systematic reviews (CRD42017074966). Some deviations from the original protocol were required in response to the material available in the reviewed articles, which was unknown at the time of planning the review. These were: (i) the wording of the research questions was improved, (ii) the review was restricted to spinal manipulative therapy (i.e. did not include extremities), and (iii) the results were analyzed depending on three categories of study subjects instead of the two we planned. Regarding the latter, it was initially planned to analyze the results depending on whether study subjects were (i) healthy or (ii) symptomatic. However, a third type of study subjects was identified, namely “subclinical neck/spinal pain” subjects. Further details on these study subjects are given in a subsequent section (see *Data analysis and synthesis*).

### Search for literature

A systematic literature search was conducted in three electronic databases: PubMed, Embase and PEDro in April 2017 (updated between January and February 2018). The search strategy was initially developed for PubMed (available in Additional file 1) and then adapted to the two other databases in collaboration with a health science research librarian. In short, the strategy was designed by associating (i) terms related to SM, for example “manipulation, spinal”, “musculoskeletal manipulations”, or “high-velocity low-amplitude spinal manipulation”, (ii) terms related to brain or brain structures, for example “brain”, “cerebrum”, or “cerebellum”, and (iii) terms related to the different ways of assessing ‘brain function’, for example “transcranial magnetic stimulation”, “electroencephalography”, or “positron-emission tomography”.

### Eligibility criteria

The eligible studies in this review had to include at least one control group, with or without random allocation. Two- or several-arm trials were accepted as well as crossover designs. These studies had to be conducted on humans, with no restriction regarding their study population such as age, sex, healthy or symptomatic subjects, or type of symptoms.

The tested intervention had to consist of manually performed, instrumentally assisted, or mechanically assisted SM. Studies with combined or concomitant therapies were excluded, as it would not be possible to separate results obtained from the SM and the other therapies. However, if all the study groups of a report were subjected to the same combined or concomitant therapies (e.g. pain medication), i.e. the only difference between the study groups being that one group was subjected to the tested intervention (e.g. pain medication AND spinal manipulation) but not the other (i.e. pain medication only), the article could be included.

The control group could be subjected to a sham procedure, an ‘inactive control’, or ‘another physical stimulus’ (other than SM). However, only studies using a sham as comparator could be considered to investigate the *effect* of SM (i.e. effect specific to SM) on the brain and could therefore be used to answer the search questions 1 to 3 of the present review. The control was considered as ‘another physical stimulus’ when it involved at least a manual contact (e.g. passive movement of a spine region, or joint preloading), or when it included other forms of manual therapies (e.g. joint mobilization, therapeutic touch). ‘Inactive control’ would consist of, for example, placing the study subject in side posture without manual contact or just resting.

Given our overall aim, the inclusion criteria were not limited to specific outcome measures or to specific measurement procedures. Studies were included if their authors stated that the outcome measures were used to assess ‘brain function’, meaning this was not necessarily expected that the outcome measures were valid or markers exclusive of ‘brain function’. This lenient criterion was chosen for two reasons: (i) some outcome measures are known also to depend on segmental activity (e.g. the V-wave) [12] and (ii) the outcome measures used by “functional neurologists” are not necessarily valid [13]. These potential issues will be discussed later in the review.

There was no restriction in relation to the date of publication of the studies but only articles in English or French were included.

### Screening

Eligibility criteria were applied twice to the titles by the first author, who also searched the reference lists of the included full texts for additional relevant studies. Thereafter, the abstracts and then the relevant full texts were read independently by two authors (ALM and CLY) to determine if they could be included in the review.

### Extraction of information

Three types of specific checklists were developed for this review relating to: main descriptive features of included articles (Tables 1, 2 and 3), methodological quality assessment (Tables 4, 5, 6, 7 and 8), and report of results (Table 9, 10 and 11). Information of interest was extracted from the Methods and Results sections only.

#### Descriptive information

Main descriptive features of the included articles were reported in three tables, one for each type of control, i.e. sham, ‘inactive’, and ‘another physical stimulus’ (see Tables 1, 2 and 3). The descriptive data were extracted from each included article independently by ALM and CLY and were later compared to minimize extraction errors.

#### Information related to methodological quality

A quality checklist was designed in order to evaluate mainly risk of bias of the type of studies included in the present review. This checklist was developed based on concepts described in the CONSORT statements [30] and on usual concepts in relation to risk of bias, such as those used by the Cochrane collaboration [31] and the scale proposed in the PEDro database [32]. The items used for the quality assessment and their rationale are described in Additional file 2.

When deemed necessary, three researchers (MAA, MS and PS), with an expertise in at least one of the outcomes used in the included studies, provided comments in relation to the methodology and technical aspects of the studies they assessed. These comments could be used to discuss the findings in relation to each research objectives. Some of these comments have been included in this report (see Tables 4, 5, 6 and 7, col.10). Each expert (MAA, MS and PS) dealt with articles within their own areas of expertise only. One of the authors (MAA), with special expertise on the types of statistical analyses used in experimental studies, reviewed all the statistical analyses. The articles were grouped by type of outcomes or families of outcomes in five methodological quality checklists of similar items (see Tables 4, 5, 6, 7 and 8). This was done in order to facilitate the overview of the comments of the experts in relation to each type of outcomes or families of outcomes. As we did not have access to experts on all the outcomes used in the included studies, comments related to methodology and technical aspects could not always be provided (as mentioned in Tables 5, 6 and 8, col.10).

Each article was independently reviewed for each methodological quality item by at least two of the authors (ALM and CLY or ALM, CLY and PS). Data were later compared to minimize extraction errors. Discrepancies were planned to be resolved by discussion between the authors.

#### Information related to the results

The outcomes of the selected studies were reported in three tables (see Tables 9, 10 and 11), one for each type of control (sham, ‘inactive’, and ‘another physical stimulus’). For each of these tables, results were reported grouped by (i) type of study subjects (healthy, symptomatic, and with “subclinical neck/spinal pain”), (ii) type of outcomes, and (iii) consecutively by year of publication.

In accordance with the recommendations of Bland and Altman (2011) [33], we planned to report only results that reflected clearly differences *between-*groups (in trials consisting of at least two separate groups of study subjects). In crossover designs, differences should be tested *between*-types of interventions. This means that results of studies that did not perform and clearly report comparisons *between*-groups or, in the case of crossover studies, failed to report *between*-types of interventions would not be taken into consideration to answer our research questions. Therefore, if authors reported only significant *within*-groups or *within*-types of interventions, without taking into account the difference between readings of the two interventions, this would be ignored.

However, our review revealed both unusual and confusing statistical reporting. We therefore decided to take into consideration also some results in a ‘benefit of the doubt’ approach, such as instances when none of the reviewers was able to decide whether the authors had, in fact, performed an appropriate *between*-groups or *between-*types of intervention analysis.

Only the primary outcomes of the included studies were considered.

### Classifying articles by their methodological quality

Each article was checked for each quality item, giving either half of a point or one point for each fulfilled item as described in Additional file 2. The quality score was arbitrarily divided into ‘acceptable’ (68 to 100% of maximum number of points), ‘medium’ (34 to 67% of maximum number of points) and ‘low’ (0 to 33% of maximum number of points), to indicate the quality of the methodological aspects mainly in relation to risk of bias of studies.

### Data analysis and synthesis

The various tables were used to report narratively the main findings in relation to our five research questions, taking into account the methodological quality of the individual studies, so that we would have more confidence in the studies of better quality than those with additional methodological deficiencies.

For each type of control, the findings were reported by type of study subjects: healthy, symptomatic, or “subclinical neck/spinal pain’” subjects. Following the definition given by the authors of the respective studies, the “subclinical neck/spinal pain” subjects appeared to us as an independent category of study subjects, neither healthy nor in pain at the time of study. Although this latter definition changes somewhat from one publication to another, study subjects were usually described as having a history of “mild intermittent spinal pain, ache or tension (subclinical spinal pain), and evidence of dysfunction in the spinal and/or pelvic joints” [24]; spinal/pelvic dysfunction referring to the chiropractic concept of ‘subluxation’ [14, 20, 25, 27, 28]. In some of these studies, these study subjects were also defined as not yet having sought treatment for their complaint [14, 15, 24, 27].

## Results

**Fig. 1:**
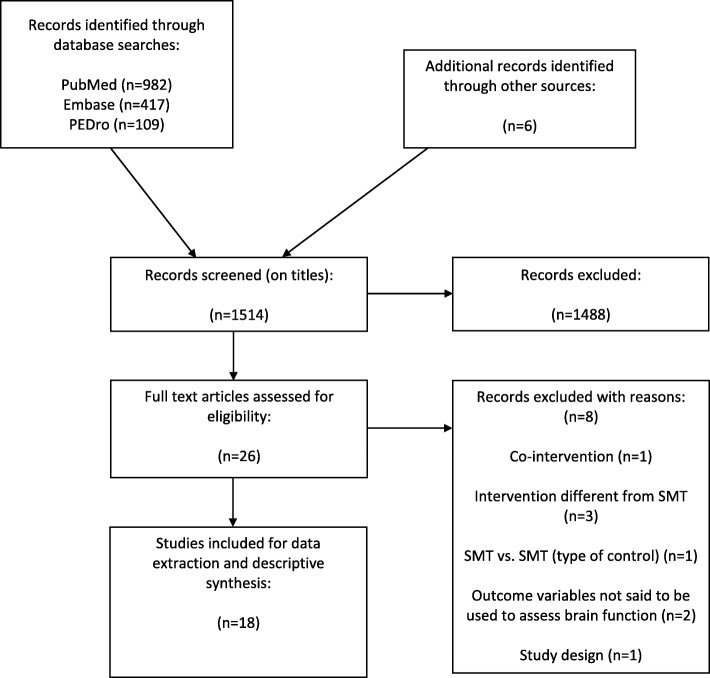
Description of the search for literature in a systematic review on the effect of spinal manipulation on ‘brain function’

Figure 1 shows a flow diagram of the study selection process. Of the 1514 initially screened articles, 18 fulfilled our inclusion criteria and were included in the review. These were published in English between 2000 and 2018. The majority of studies (*n* = 10), all on “subclinical neck/spinal pain” subjects, were conducted by research teams that included one specific author [14, 15, 20, 21, 23–28].

All articles reported an ethics approval from an ethics committee or from a review board, with or without an identification number of the application and approval. As for conflict of interest, 11 studies declared to have none [8, 9, 14–16, 18, 21, 22, 24, 26, 28], whereas the issue of conflict was not mentioned at all in the others [17, 19, 20, 23, 25, 27, 29].

### Description of studies (*n* = 18)

Detailed descriptive information of each study is available in Tables 1, 2 and 3 and briefly summarized below.

**Table 1:** Description of three studies included in a systematic review on the effect of spinal manipulation on ‘brain function’, comparing spinal manipulation to a sham intervention

1st author Yr Ref	Design	Type of study subjects	Number of study subjects (males/females)	-Age (range) -Mean	-Type of spinal manipulation -Type of control -Sham	How was cerebral activity measured?	When was cerebral activity measured?	Clinical outcomes assessed (measurement tool and time of assessment)
Spars 2017 [9]	Randomized controlled trial	Symptomaic: volunteers (unknown origin) with mechanical neck pain < 6 weeks of duration	24 (4 / 8) manipulation group / (4 / 8) sham group	-? /? -36 manipulation group / 40 sham group	-HVLA midthoracic (X1) -‘No’ control -Sham: similar positioning of the subject and investigator’s hands which were placed across the skin with minimal pressure (to mimic the HVLA procedure)	Blood oxygenation level-dependent signal (in response to noxious stimuli)	Before After: immediately	Pain intensity (11-point numerical pain rating scale) (before spinal manipulation or sham procedures and after the final fMRI)
Lelic 2016 [14]	Crossover controlled trial (order of interventions randomized)	“Subclinical neck/spinal pain”: volunteers (origin unknown) with recurrent spinal ache, pain or stiffness and evidence of spinal dysfunction but who did not yet sought treatment for this and pain free at the time of the study.	19 (9 / 10)	-? -26	-HVLA (where needed, in any spine level or sacroiliac joints, nb unknown _ may be at several levels) -‘No’ control -Sham: passive and active movements of the head, spine, and body, similar to what was done for HVLA intervention, without loading and thrust	SEP amplitudes: N30 peaks Strength of brain sources: contralateral somatosensory cortex, prefrontal cortex, cingulate cortex, and bilateral secondary somatosensory cortex	Before After: exact time unknown	None
Baarbé 2018 [15]	Randomized controlled trial	“Subclinical neck/spinal pain”: volunteers (unknown origin) with recurrent mild neck pain and muscle tension, but minimal acute pain on the day of testing and who never sought treatment for this neck complains.	27 (6 / 8) intervention group / (5 / 8) sham group	-18–27 intervention group / 19–24 sham group -21 (for both groups)	-HVLA cervical (X2 to 4 per subject) -‘No’ control -Sham: neck gently moved into lateral flexion and rotation in a similar manner to the actual neck manipulation, without applying the HVLA thrust	Cerebellar inhibition	Before After: exact time unclear (said to be immediately after motor acquisition task, i.e. cerebellar inhibition was re-measured about 20 min after spinal manipulation)	None

Articles are presented by (i) type of study subjects, i.e. symptomatic or “subclinical neck/spinal pain” subjects, and (ii) consecutively by year of publication

*fMRI* Functional magnetic resonance imaging, *HVLA* High velocity low amplitude, *nb* Number, *SEP* Somatosensory evoked potential

**Table 2:** Description of eight studies included in a systematic review on the effect of spinal manipulation on ‘brain function’, comparing spinal manipulation to an inactive control

1st author Yr Ref	Design	Type of study subjects	Number of study subjects (males/females)	-Age (range) -Mean	-Type of spinal manipulation -Type of control	How was cerebral activity measured?	When was cerebral activity measured?	Clinical outcomes assessed (measurement tool and time of assessment)
Kelly 2000 [16]	Randomized controlled trial	Healthy: volunteer chiropractic students with evidence of upper cervical “subluxation”.	36 (9 / 9) intervention group / (11 / 7) control group	-20-37 (both groups) -24 (both groups)	-Toggle (X1) -Control: 2 min of resting	Mental rotation reaction-time task	Before After: exact time unknown	None
Dishman 2002 [17]	Non-randomized controlled trial	Healthy: healthy college students, volunteers	24 (? /?) (repartition in each group not reported)	-? /? -25 intervention group / 27 control group	-HVLA L5-S1 (X1) -Control: side posture positioning without lower limb flexion, truncal torque, or manual contact	MEP amplitudes	Before After: -immediately (20 to 120 s) -5 min −10 min	None
Dishman 2008^a^ [18]	Randomized controlled trial	Healthy: healthy chiropractic students, volunteers	72 (21 / 5) intervention group / (15 / 8) control 1 / (14 /9) control 2	-? (3 groups, said to be between their 20s and 30s) -? (3 groups)	-HVLA L5-S1 (X1) -Control 1: L5-S1 preloading -Control 2: side posture positioning	MEP amplitudes	Before (10 MEP recorded during 100 s) After: immediately (10 MEP recorded during 100 s)	None
Fryer 2012 [16]	Crossover controlled trial (order of interventions randomized)	Healthy: healthy university students, volunteers	14 (10 / 4)	-18-50 -23	-HVLA L5-S1 (X2 to 4) -Control: bilateral side-posture positioning without truncal torque, or manual contact	MEP latencies and amplitudes Silent periods	Before After: exact time unknown (according to the Discussion approximately 10 min after)	None
Ogura 2011 [19]	Crossover controlled trial (order of interventions “counterbalanced”)	Symptomatic: volunteers, recruited at the local university, with mechanical cervical pain and shoulder stiffness.	12 (12 / 0)	-21–40 -28	-Instrumentally assisted manipulation (location and nb of spinal levels adjusted unknown) -Control: 20 min of resting	Regional cerebral metabolic rate (rate of glucose consumption)	No before measurementAfter: between 35 to 55 min post- intervention or resting	-Stress Response Scale (immediately after interventions) -European Organization for Research and Treatment of Cancer Quality of Life Questionnaire-Core 30 (immediately after interventions) -Pain intensity (visual analogue scale) (before and immediately after spinal manipulation, not before- after-20 min of resting)
Inami 2017 [8]	Crossover controlled trial (order of interventions randomized)	Symptomatic: volunteers (unclear origin – probably the same as Ogura et al. 2011) with mechanical cervical pain and shoulder stiffness.	21 (21 / 0)	-? -26	-Instrumentally assisted manipulation (where needed, anywhere at the spine, sacroiliac joints and/or scapulae, mean of 8 per subject) -Control: 20 min of resting	Regional cerebral metabolic rate (rate of glucose consumption)	No before measurement After: between 35 min to 1.05 h. post-intervention or resting	Pain intensity (visual analogue scale) (before and immediately after spinal manipulation, and before and after 20 min of resting, only for 9/21 subjects)
Haavik-Taylor 2007a^a^ [20]	Crossover controlled trial (order of interventions randomized)	“Subclinical neck/spinal pain”: volunteers (unknown origin) with a history of recurring neck pain or stiffness and with evidence of cervical spinal dysfunction, pain free at the time of the study.	13 (5 / 8)	-22-45 -31	-HVLA cervical (X2 to 3 per subject) -Control 1: passive head movement without loading and thrust -Control 2: nothing	MEP amplitudes CSP durations	Before After: -within 0–10 min -within 10–20 min -within 20–30 min	None
Haavik-Taylor 2010b [21]	Crossover controlled trial (order of interventions randomized)	“Subclinical neck/spinal pain”: student and university staff members, volunteers, with reoccurring neck problems and evidence of cervical spine dysfunction, pain free at the time of the study.	11 (4 / 7)	-22-40 -29	-HVLA cervical (nb unknown, may be at several levels) + 20 min of typing task -Control: 20 min of typing task only	SEP MU/M + U peak ratios: -P14-N18 complex -Parietal N20 (N20-P25 complex) -Frontal N30 (P22-N30 complex)	Before After: exact time unclear (said to be immediately after HVLA+ 20 min of typing task or after 20 min typing task only, but also said to be within 25 min post interventions, i.e. possibly within 45 min after spinal manipulation)	None

Articles are presented by (i) type of study subjects, i.e. healthy, symptomatic or “subclinical neck/spinal pain” subjects, (ii) type of outcomes or family of outcomes, and (iii) consecutively by year of publication

*CSP* Cortical silent period, *HVLA* High-velocity low-amplitude, *MEP* Motor evoked potential, *nb* Number, *SEP* Somatosensory evoked potential

^a^Article presented in Tables 2 and 3

**Table 3:** Description of nine studies included in a systematic review on the effect of spinal manipulation on ‘brain function’, comparing spinal manipulation to another physical stimulus

1st author Yr Ref	Design	Type of study subjects	Number of study subjects (males/females)	-Age (range) -Mean	-Type of spinal manipulation -Type of control	How was cerebral activity measured?	When was cerebral activity measured?	Clinical outcomes assessed (measurement tool and time of assessment)
Dishman 2008^a^ [18]	Randomized controlled trial	Healthy: healthy chiropractic students, volunteers.	72 (21 / 5) intervention group / (15 / 8) control 1 / (14 /9) control 2	-? (3 groups, said to be between their 20s and 30s) -? (3 groups)	-HVLA L5-S1 (X1) -Control 1: L5-S1 preloading -Control 2: side posture positioning	MEP amplitudes	Before (10 MEP recorded during 100 s) After: immediately (10 MEP recorded during 100 s)	None
Gay 2014 [22]	Randomized controlled trial	Symptomatic: volunteers from a previous clinical trial, recruited at the local university, hospital and surrounding community, who completed an exercise-injury protocol to induce myalgia in the low back.	24 (1 / 5) manipulation group / (1 / 7) mobilization group / (5 / 5) therapeutic touch group (7 / 17)	-? /? /? (required to be between 18 and 44) −21 manipulation group / 21 mobilization group / 23 therapeutic touch group	-HVLA (X1, probably in the lumbar spine) -Control 1: grade III lumbar spinal mobilization -Control 2: therapeutic touch (light pressure with a contact to the sacroiliac joints)	Functional connectivity	Before After: immediately	Pain intensity (101-point numerical rating scale) (before and immediately after in each group)
Haavik-Taylor 2007a^a^ [20]	Crossover controlled trial (order of interventions randomized)	“Subclinical neck/spinal pain”: volunteers (unknown origin) with a history of recurring neck pain or stiffness and with evidence of cervical spinal dysfunction, pain free at the time of the study.	13 (5 / 8)	−22-45 −31	-HVLA cervical (X2 to 3 per subject) -Control 1: passive head movement without loading and thrust -Control 2: nothing	MEP amplitudes CSP durations	Before After: -within 0–10 min -within 10–20 min -within 20–30 min	None
Haavik-Taylor 2008 [23]	Crossover controlled trial (order of interventions randomized)	“Subclinical neck/spinal pain”: adults (unknown origin) with a history of reoccurring neck pain or stiffness and with evidence of cervical spinal dysfunction, pain free at the time of the study.	12 (7 / 5)	−19-45 −27	-HVLA cervical (nb unknown, may be at several levels) -Control: passive head movement without loading and thrust	MEP amplitudes CSP durations SICI SICF	Before After: exact time unknown	None
Haavik 2016 [24]	Crossover controlled trial	“Subclinical neck/spinal pain”: volunteers (unknown origin) with a history of spinal symptoms and with evidence of spinal and/or pelvic dysfunction but who did not yet sought treatment for this and pain free at the time of the study.	12 (?)	-? −28	-HVLA cervical (nb unknown, may be at several levels) -Control: passive head movement without loading and thrust	MEP amplitudes Slope of the steepest part of the curve (k) Stimulus intensity required to obtain a response that is 50% of the max (S_50_)	Before After: exact time unknown	None
Haavik-Taylor 2007b [25]	Two groups “pseudo-randomized” trial	“Subclinical neck/spinal pain”: volunteers (origin unknown) with reoccurring neck problems and evidence of cervical spine dysfunction, pain free at the time of the study.	24 (7 / 5) intervention group / (4 / 8) control group	−20-53 intervention group / 21–35 control group −30 intervention group / 27 control group	-HVLA cervical (X2 to 3 per subject) -Control: passive head movement without loading and thrust	SEP latencies and amplitudes: P14–18 complex, N20 (P14-N20 and N20-P27) and N30 (P22-N30) peaks	Before After: -within 0–10 min -within 10–20 min -within 20–30 min	None
Haavik-Taylor 2010a [26]	Crossover controlled trial (order of interventions randomized)	“Subclinical neck/spinal pain”: volunteers (origin unknown) with reoccurring neck problems and evidence of cervical spine dysfunction, pain free at the time of the study.	13 (5 / 8)	−18-40 −28	-HVLA cervical (nb unknown, may be at several levels) -Control: passive head movement without loading and thrust	SEP MU/M + U peak ratios: P14-N18 complex, N20-P25 complex, and P22-N30 complex ratios	Before After: within 25 min	None
Niazi 2015 [27]	Crossover controlled trial (order of interventions randomized)	“Subclinical neck/spinal pain”: volunteers (origin unknown) with recurring, intermittent low-grade spinal pain, ache, or tension, with evidence of spine dysfunction, but which did not sought treatment for this problem and are pain free at the time of the study.	10 (10 / 0)	-? (required to be between 18 and 40) −28	-HVLA (where needed, in any spine level or sacroiliac joints, nb unknown _ may be at several levels) -Control: passive and active movements of the subject’s head, spine, and body into the manipulation setup positions, without loading and thrust	V-wave amplitude	Before After: exact time unknown	None
Christiansen 2018 [28]	Crossover controlled trial (order of interventions randomized)	“Subclinical neck/spinal pain”: elite Taekwondo athletes, from the Auckland area, with “subclinical spinal pain” and evidence or spine dysfunction, pain free at the time of the study.	12 (6 / 6)	-? (required to be between 17 and 50) −25	-HVLA (where needed, in any spine level or sacroiliac joints, nb unknown _ may be at several levels) -Control: passive and active movements of the subject’s head and spine into the manipulation setup positions, without loading and thrust	V-wave amplitude	Before After: -immediately −30 min −60 mins	None

Articles are presented by (i) type of study subjects, i.e. healthy, symptomatic or “subclinical neck/spinal pain” subjects, (ii) type of outcomes or family of outcomes, and (iii) consecutively by year of publication

*CSP* Cortical silent period, *HVLA* High-velocity low-amplitude, *MEP* Motor evoked potential, *nb* Number, *SEP* Somatosensory evoked potential, *SICF* Short interval intra-cortical inhibition, *SICI* Short interval intra-cortical facilitation, *SM* Spinal manipulation

^a^Article presented in Tables 2 and 3

The size of the study samples of the 18 included studies ranged from 10 to 72. Ten were conducted on “subclinical neck/spinal pain” subjects (Tables 1, 2 and 3), four on pain free healthy subjects (Tables 2 and 3), and four on symptomatic subjects (Tables 1, 2 and 3), including one on subjects with experimentally induced low-back myalgia [22]. In five of the ten studies considered in the present review, as conducted on “subclinical neck/spinal pain” subjects, the study subjects were not explicitly described as such [20, 21, 23, 25, 26]. However, the description provided by their authors clearly referred to the definition of “subclinical neck/spinal pain” subjects [24].

All the included studies were controlled trials, including two to three experimental groups, most of them with a random allocation (*n* = 14) and mostly conducted using a crossover design (*n* = 10). Only one study reported the dates and duration of data collection [9].

Most of the included articles investigated high-velocity low-amplitude SM, whereas three investigated instrumentally or mechanically assisted techniques. The area where SM was provided varied across studies to include all areas of the spine, whereas one study did not indicate where [19]. In most of them SM was provided ‘where deemed necessary’.

Most studies used as a control group some passive type of procedure, considered in the present systematic review as ‘another physical stimulus’, or used a completely ‘inactive control’, whereas three attempted to use some types of sham comparators [9, 14, 15]. One study compared SM to two other manual therapies, i.e. spinal mobilization and therapeutic touch of the lumbosacral area [22].

The outcomes of all these studies were either described as *reflecting* some type of ‘brain function(s)’ or as *suggesting* some type of ‘brain function(s)’, meaning that some outcomes could also reflect, for example, neurophysiological changes at a segmental level (e.g. V-wave, motor evoked potentials, or cortical silent periods) [12, 34]. The outcome measures and measurement tools used in the selected studies are briefly described in Additional file 3. In two studies, outcomes were assessed only *after* intervention or control, presumably for ethical reasons [8, 19]. As can be seen in Tables 1, 2 and 3, in all other studies outcomes were assessed *before* and *after* intervention at various time points. However, six studies did not specify the time of re-assessment at all and four did not report it clearly.

The four studies conducted on symptomatic subjects described in their Methods section that they also assessed clinical outcomes, mainly pain intensity (see Tables 1, 2 and 3) [8, 9, 19, 22]. However, only two of them had as one of their research objectives to investigate whether a relation exists between potential neurophysiological changes and pain intensity changes after intervention, and none of these two assessed this against a sham intervention [19, 22].

### Data extraction (*n* = 18)

The data extraction process was relatively problem free with only few exceptions. These concerned some articles in which the statistics and/or results sections were unclear [9, 14, 21–23, 26, 27]. This was resolved through multiple discussions. In addition, experts’ opinions were sought in these areas and the experts (MAA, MS and PS) also reported difficulty to interpret some of the studies [9, 14, 21, 23, 26].

### Data synthesis: methodological quality of the studies (*n* = 18)

The level of methodological quality was generally ‘low’ (*n* = 7) or ‘medium’ (*n* = 8), except for three articles that were considered to be of ‘acceptable’ quality (see Table 12 for a summary). The most frequently encountered methodological weaknesses were: (i) the success of the blinding of the subjects was uncertain or unsuccessful (the three ‘*effect’* studies), (ii) no clear reporting whether the study was conducted on naïve subjects (most of the ‘*differences in outcome’* studies), (iii) no reporting whether the assessor was blinded to treatment group (most studies), and (iv) no reporting whether the person who analyzed the data was blinded to treatment group (most studies). In addition, the experts (MAA, MS and PS) sometimes commented on unusual procedures (for detailed information see Tables 4, 5, 6, 7 and 8 col.10).

**Table 4:** Quality items and score of one study using a reaction-time task included in a systematic review on the effect of spinal manipulation on ‘brain function’

1st Author Yr of publication Ref	-Were study subjects in sham controlled studies reported to be blind? (Yes / No / Unclear) -If yes / unclear, was the blinding tested for success? (Yes / No) -If yes, was it successful? (Yes / No)	-Were study subjects in studies with control group reported to be naive? (Yes / No / Unclear) -Was the origin of the subjects reported (Yes / No) -If yes, does it allow to exclude any interest? (Yes / No / Unclear)	Were study subjects reported to have been randomly allocated to study groups? (Yes / No / Unclear)	Were study groups comparable in relation to symptoms when studying symptomatic subjects (duration and pain intensity) (NA when cross-over study design)? (Yes / No)	Were the intervention and control(s) well described (at least where and how)? (Yes / No)	Was the assessor reported to be blind to group allocation? (Yes / No)	Were losses and exclusions of study subjects reported or obvious in result section (including in tables or graphs)? (Yes / No / Unclear)	Was the person who statistically analyzed the data reported to be blind to group allocation? (Yes / No)	Comments by the technical experts (i) on the statistical analysis, and (ii) in relation to the methodology and/or technical aspects
Quality score (risk of bias, also including an external validity criteria) and classification									
Kelly 2000 [29]		-Yes (but in relation to the outcome) -Yes -No							1: -The authors used a Student *t* tests to compare means instead of using a mixed-model ANOVA, followed by post-hoc tests if needed. -The authors did not study how RT (for correct answers) varied with angle, which is the main analysis conducted in the literature on such data. Therefore, without such a (usually linear) trend analysis it is not possible to understand if the overall mean effect observed by the authors is due to a change in slope (reflecting a change in processing speed) or in intercept (reflecting a change in stimulus encoding). 3: -Between-group difference pre-post significant only with one-sided t-test. -The between-group difference pre-post is not reported for the simple RT task but it seems that a contribution of the simple RT to the RT of the complex task cannot be excluded. -Unclear whether errors were also counted.
3.5/6 (58%) medium	NA	= Unclear 0.5 pt	Yes 1 pt	NA (healthy subjects)	-Yes 0.5 pt -Yes 0.5 pt	No 0 pt	Yes 1 pt	No 0 pt	

*NA* Not applicable, *RT* Rreaction time

**Table 5:** Quality items and scores of seven studies using transcranial magnetic induced outcome measures included in a systematic review on the effect of spinal manipulation on ‘brain function’

1st Author Yr of publication Ref	-Were study subjects in sham controlled studies reported to be blind? (Yes / No / Unclear) -If yes / unclear, was the blinding tested for success? (Yes / No) -If yes, was it successful? (Yes / No)	-Were study subjects in studies with control group reported to be naive? (Yes / No / Unclear) -Was the origin of the subjects reported (Yes / No) -If yes, does it allow to exclude any interest? (Yes / No / Unclear)	Were study subjects reported to have been randomly allocated to study groups? (Yes / No / Unclear)	Were study groups comparable in relation to symptoms when studying symptomatic subjects (duration and pain intensity) (NA when cross-over study design)? (Yes / No)	Were the intervention and control(s) well described (at least where and how)? (Yes / No)	Was the assessor reported to be blind to group allocation? (Yes / No)	Were losses and exclusions of study subjects reported or obvious in result section (including in tables or graphs)? (Yes / No / Unclear)	Was the person who statistically analyzed the data reported to be blind to group allocation? (Yes / No)	Comments by the technical experts (i) on the statistical analysis, and (ii) in relation to the methodology and/or technical aspects
Quality score (risk of bias, also including an external validity criteria) and classification									
Dishman 2002 [17]		-No -Yes -No							2: -MEP methodology does not correspond to standard: no motor threshold, no force control, and lack of random intervals between stimulus -The coil positioning seems not appropriate to lower leg MEPs.
2.5/6 (42%) medium	NA	= No 0 pt	Unclear ("counterbalanced") 0.5 pt	NA (healthy subjects)	-Yes 0.5 pt -Yes 0.5 pt	No 0 pt	Yes 1 pt	No 0 pt	
Haavik-Taylor 2007a [20]		-No -No -NA							1: -The authors stated having used *planned comparisons* instead of post hoc analysis in order to minimize Type 1 error. However,*planned comparison*s do not minimize Type1 error. -They mention running a one-way repeated measures ANOVA with the factor “intervention”. However, the degrees of freedom of the F for the result clearly show that authors treated “intervention” as a between-subjects factor, which is not correct.
2/6 (33%) low	NA	= No 0 pt	Yes 1 pt	NA (SCP subjects / cross-over)	-Yes 0.5 pt -Yes 0.5 pt	No 0 pt	No 0 pt	No 0 pt	
Dishman 2008 [18]		-No -Yes -No							2: -MEP methodology is not standard: lack of precise motor threshold, and lack of random intervals between stimulus. -Fig. 1C indicates an inhibition in the time interval prior to SM, which may be responsible for significant differences and relative increase of amplitude after SM.
2/6 (33%) low	NA	= No 0 pt	Yes 1 pt	NA (healthy subjects)	-Yes 0.5 pt -Yes 0.5 pt	No 0 pt	No 0 pt	No 0 pt	
Haavik-Taylor 2008 [23]		-No -No -NA							1: -The authors mention running 2-way ANOVAs for repeated measures with the factors “muscle” and “intervention” were applied to compare the effects of SM on the two different upper limb muscles. However, the degrees of freedom of the F for the results clearly show that authors treated the two factors between-subjects, which is not correct. -They use *t* tests instead of post-hoc test for testing pairwise comparisons subsequent to the ANOVA. 2: The conclusions are farfetched as assumptions and deduction are made which cannot not be backed by the results.
2/6 (33%) low	NA	= No 0 pt	Yes 1 pt	NA (SCP subjects / cross-over)	-Yes 0.5 pt -Yes 0.5 pt	No 0 pt	No 0 pt	No 0 pt	
Fryer 2012 [16]		-No -Yes -No							2: The coil positioning seems not appropriate to lower leg MEPs.
2.5/6 (42%) medium	NA	= No 0 pt	Yes 1 pt	NA (healthy subjects)	-Yes 0.5 pt -Yes 0.5 pt	No 0 pt	Unclear 0.5 pt	No 0 pt	
Haavik 2016 [24]		-Unclear (most subjects were “novice to chiropractic”) -No -NA							2: -The recruitment curves lack measure of variance. -Feedback from background EMG is lacking, which is a conceptual concern and could explain observed increased in amplitudes.
2/6 (33%) low	NA	= No 0 pt	No 0 pt	NA (SCP subjects / cross-over)	-Yes 0.5 pt -Yes 0.5 pt	No 0 pt	Yes 1 pt	No 0 pt	
Baarbé 2018 [15]	-Yes -No -NA								None in relation to statistics *No expert was available in relation to the technical aspects of this outcome measure*
3.5/6 (58%) medium	= Unclear 0.5 pt	NA	Yes 1 pt	NA (SCP subjects)	-Yes 0.5 pt -Yes 0.5 pt	No 0 pt	Yes 1 pt	No 0 pt	

*EMG* Electromyography, *MEP* Motor-evoked potential, *NA* Not applicable, *SCP* “subclinical neck/spinal pain”, *SM* Spinal manipulation

**Table 6:** Quality items and scores of four studies using outcome measures in relation to somatosensory-evoked potentials included in a systematic review on the effect of spinal manipulation on ‘brain function’

1st Author Yr of publication Ref	-Were study subjects in sham controlled studies reported to be blind? (Yes / No / Unclear) -If yes / unclear, was the blinding tested for success? (Yes / No) -If yes, was it successful? (Yes / No)	-Were study subjects in studies with control group reported to be naive? (Yes / No / Unclear) -Was the origin of the subjects reported (Yes / No) -If yes, does it allow to exclude any interest? (Yes / No / Unclear)	Were study subjects reported to have been randomly allocated to study groups? (Yes / No / Unclear)	Were study groups comparable in relation to symptoms when studying symptomatic subjects (duration and pain intensity) (NA when cross-over study design)? (Yes / No)	Were the intervention and control(s) well described (at least where and how)? (Yes / No)	Was the assessor reported to be blind to group allocation? (Yes / No)	Were losses and exclusions of study subjects reported or obvious in result section (including in tables or graphs)? (Yes / No / Unclear)	Was the person who statistically analyzed the data reported to be blind to group allocation? (Yes / No)	Comments by the technical experts (i) on the statistical analysis, and (ii) in relation to the methodology and/or technical aspects
Quality score (risk of bias, also including an external validity criteria) and classification									
Haavik-Taylor 2007b [25]		-No -No -NA							1: -No report of the testing of the normality of the data distribution. -To minimize Type 1 error, post hoc tests would be appropriate (instead of planned comparisons). -No between group comparison was performed. *No expert was available in relation to the technical aspects of this outcome measure*
2/6 (33%) low	NA	=No 0 pt	Unclear ("pseudorandomized") 0.5pt	NA (SCP subjects)	-Yes 0.5pt -Yes 0.5pt	No (but data were coded by an independent person to reduce any bias during analysis) 0.5pt	No 0 pt	No 0 pt	
Haavik-Taylor 2010a [26]		-No -No -NA							1: Both parametric and nonparametric results on the same data are reported. Usually, either data are normally distributed and parametric tests can be used or data are not normally distributed and non-parametric tests must be used. *No expert was available in relation to the technical aspects of this outcome measure*
2.5/6 (42%) medium	NA	= No 0 pt	Yes 1pt	NA (SCP subjects / cross-over)	-Yes 0.5 pt -Yes 0.5 pt	No (idem Haavik-Taylor 2007a) 0.5pt	No 0 pt	No 0 pt	
Haavik-Taylor 2010b [21]		-No -Yes -Unclear (“students and staff population at the University of Auckland”)							1: See comments in relation to Haavik-Taylor 2010a *No expert was available in relation to the technical aspects of this outcome measure*
2.5/6 (42%) medium	NA	= No 0 pt	Yes 1 pt	NA (SCP subjects / cross-over)	-Yes 0.5 pt -Yes 0.5 pt	No (idem Haavik-Taylor 2007b) 0.5 pt	No 0 pt	No 0 pt	
Lelic 2016 [14]	-Unclear (said to be naïve) -Yes -No (sham intervention was discovered as such by most of the subjects)								1: Unusual reporting of statistics: no report of which were the experimental factors and how they were treated (but probably pre/post was treated within-subjects and interventions as between-subjects), and of the detailed results for the *F* tests of the ANOVA. *No expert was available in relation to the technical aspects of this outcome measure*
2.5/6 (42%) medium	= No 0 pt	NA	Yes 1 pt	NA (SCP subjects / cross-over)	-Yes 0.5 pt -No 0 pt	No 0 pt	Yes 1 pt	No 0 pt	

*NA* Not applicable, *SCP* “subclinical neck/spinal pain”

**Table 7:** Quality items and scores of four studies using neuroimaging outcome measures included in a systematic review on the effect of spinal manipulation on ‘brain function’

1st Author Yr of publication Ref	-Were study subjects in sham controlled studies reported to be blind? (Yes / No / Unclear) -If yes / unclear, was the blinding tested for success? (Yes / No) -If yes, was it successful? (Yes / No)	-Were study subjects in studies with control group reported to be naive? (Yes / No / Unclear) -Was the origin of the subjects reported (Yes / No) -If yes, does it allow to exclude any interest? (Yes / No / Unclear)	Were study subjects reported to have been randomly allocated to study groups? (Yes / No / Unclear)	Were study groups comparable in relation to symptoms when studying symptomatic subjects (duration and pain intensity) (NA when crossover study design)? (Yes / No)	Were the intervention and control(s) well described (at least where and how)? (Yes / No)	Was the assessor reported to be blind to group allocation? (Yes / No)	Were losses and exclusions of study subjects reported or obvious in result section (including in tables or graphs)? (Yes / No / Unclear)	Was the person who statistically analyzed the data reported to be blind to group allocation? (Yes / No)	Comments by the technical experts (i) on the statistical analysis, and (ii) in relation to the methodology and/or technical aspects
Quality score (risk of bias, also including an external validity criteria) and classification									
Ogura 2011 [19]		-No -Yes -Unclear (recruited on the campus of Tohoku University)							1: The extent the threshold for the voxel cluster size was defined as “10 to 50 voxels minimum”. The purpose of this varying threshold is unclear. 3: Lenient statistical threshold: Z = 3, extent threshold; 10 voxels.
2/6 (33%) low	NA	= No 0 pt	Unclear (“counterbalanced”) 0.5 pt	NA (cross-over)	-No 0 pt -Yes 0.5 pt	No 0 pt	Yes 1 pt	No 0 pt	
Inami 2017 [8]		-No -No -NA							1: The phrasing “(e.g., 10 voxels minimum)” suggests again (see the comment in relation to Ogura 2011) that this threshold was not fixed.
2/6 (33%) low	NA	= No 0 pt	Yes 1 pt	NA (cross-over)	-Yes 0.5 pt -Yes 0.5 pt	No 0 pt	No 0 pt	No 0 pt	
Gay 2014 [22]		-No -No -Unclear (recruited from the campus of the University of Florida and UF Health Hospital and the local community)							1: -Authors “corrected for the number of separate RM-ANOVAs conducted across the 120 ROI-to-ROI pairs by using a *p* value less than .01 as significant.” (p.618). This threshold (*p* = 0.01) correction for multiple comparisons is not conservative enough. -There was neither between-groups statistical test at “pre”, nor at “post”. 3: Lenient statistical threshold: *p* = 0.01 with 120 comparisons.
5/7 (71%) acceptable	NA	= No 0 pt	Yes 1 pt	Yes 1 pt	Yes 0.5 pt -Yes 0.5 pt	Yes 1 pt	Yes 1 pt	No 0 pt	
Sparks 2017 [9]	-Yes -No -NA								1: The authors used an alpha = 0.01 threshold for the fMRI analysis. It is not conservative enough in my opinion (as discussed by Eklund et al. 2015, and Lieberman & Cunningham 2009). 3: -Unclear whether statistical threshold applied across the whole brain or just for the region of interest. -It is unclear how the region of interest was defined
5.5/7 (79%) acceptable	= Unclear 0.5 pt	NA	Yes 1 pt	Yes 1 pt	-Yes 0.5 pt -Yes 0.5 pt	Yes 1 pt	Yes 1 pt	No 0 pt	

*fMRI* functional magnetic resonance imaging, *NA* Not applicable

**Table 8:** Quality items and scores of two studies using V-wave as outcome measures included in a systematic review on the effect of spinal manipulation on ‘brain function’

1st Author Yr of publication Ref	-Were study subjects in sham controlled studies reported to be blind? (Yes / No / Unclear) -If yes / unclear, was the blinding tested for success? (Yes / No) -If yes, was it successful? (Yes / No)	-Were study subjects in studies with control group reported to be naive? (Yes / No / Unclear) -Was the origin of the subjects reported (Yes / No) -If yes, does it allow to exclude any interest? (Yes / No / Unclear)	Were study subjects reported to have been randomly allocated to study groups? (Yes / No / Unclear)	Were study groups comparable in relation to symptoms when studying symptomatic subjects (duration and pain intensity) (NA when cross-over study design)? (Yes / No)	Were the intervention and control(s) well described (at least where and how)? (Yes / No)	Was the assessor reported to be blind to group allocation? (Yes / No)	Were losses and exclusions of study subjects reported or obvious in result section (including in tables or graphs)? (Yes / No / Unclear)	Was the person who statistically analyzed the data reported to be blind to group allocation? (Yes / No)	Comments by the technical experts (i) on the statistical analysis, and (ii) in relation to the methodology and/or technical aspects
Quality score (risk of bias, also including an external validity criteria) and classification									
Niazi 2015 [27]		-No -No -NA							None in relation to statistics *No expert was available in relation to the technical aspects of this outcome measure*
2.5/6 (42%) medium	NA	= No 0 pt	Yes 1 pt	NA (SCP subjects)	-Yes 0.5 pt -No 0 pt	No 0 pt	Yes 1 pt	No 0 pt	
Christiansen 2018 [28]		-No -Yes -Yes							None in relation to statistics *No expert was available in relation to the technical aspects of this outcome measure*
5/6 (83%) acceptable	NA	= Unclear 0.5 pt	Yes 1 pt	NA (SCP subjects)	-Yes 0.5 pt -No 0 pt	Yes 1 pt	Yes 1 pt	Yes 1 pt	

*NA* Not applicable, *SCP* “subclinical neck/spinal pain”

In two of the studies it was clear that the authors did not report having performed a *between-*group analysis [20, 25]. Thus their results were not taken into account for our five research questions, and were therefore not reported in Tables 10 and 11. Another study did appear to compare the outcomes of SM on two different hand muscles rather than to compare the effect of SM to a control intervention (see Table 5 col.10) [23]. This article was therefore not reported in Table 11.

Also, three studies did not report results in relation to all the statistical *between*-group comparisons that they stated in their respective Methods sections that they would do [14, 22, 27]. For these studies, only the results from *between*-group comparisons, if present, were therefore reported.

A total of 13 studies were finally used to answer our five research questions. One of these studies appears in two of the three results tables (Tables 10 and 11) [18].

### Data synthesis: answers to research questions (*n* = 13)

#### Sham controlled studies (Table 9), i.e. ‘*effect’* studies (*n* = 3)

Only three studies used a sham comparator and were therefore considered as potentially able to provide answers to the research questions 1 to 3 [9, 14, 15]. However, in two of these the credibility of the sham is unclear [9, 15], and in the third, the sham was recognized as such by most of the study subjects [14]. Two were considered of ‘medium’ methodological quality [14, 15] and one of ‘acceptable’ methodological quality [9] (see Table 12). These studies, reporting on symptomatic subjects or on “subclinical neck/spinal pain” subjects, investigated the potential *effect* of SM on ‘brain function’ by using three different outcome measures, which did not allow us to compare their respective results (Tables 9, 10 and 11).

**Table 9:** Results from three studies included in a systematic review on the effect of spinal manipulation on ‘brain function’, comparing spinal manipulation to a sham intervention

1st Author Year Ref	Type of study subjects	Outcome variable	Was a statistically significant difference between groups observed?	Was there a relationship between brain changes and any clinical outcome?	Time of assessment	Quality classification
Sparks 2017 [9]	Symptomatic (mechanical neck pain < of 6 weeks of duration)	Blood oxygenation-level dependent signal (in response to noxious stimuli)	Yes (*p* < .05) Statistically significant increase of activation in the insular and sensorimotor cortices post-SM compared to control; and in the anterior and posterior cingulate, supplementary motor area, and precentral gyrus post-control compared to SM	Pain intensity assessed but no relationship tested	Immediately after	Acceptable
Lelic 2016 [14]	“Subclinical neck/spinal pain”	N30 somatosensory evoked potential peak amplitudes	Yes (significant post-intervention difference *between-*groups reported but without inclusion of the corresponding *p*-value and mention of the statistical threshold for significance) Statistically significant decrease post-SM (*p* = .02) but no statistically significant changes post-control (*p* = .4)	No clinical outcome included	Not reported	Medium
Baarbé 2018 [15]		Cerebellar inhibition	Yes (*p* < .001) Statistically significant reduce post-SM compared to control	No clinical outcome included	Unclear (according to Fig. 1 immediately after the motor acquisition task, i.e. about 20 min after intervention)	Medium

Results are reported (i) grouped by type of study subjects (symptomatic or with “subclinical neck/spinal pain”), and (ii) consecutively by year of publication

*SM* Spinal manipulation

**Table 10:** Results from seven studies included in a systematic review on the effect of spinal manipulation on ‘brain function’, comparing spinal manipulation to an inactive control

1st Author Year Ref	Type of study subjects	Outcome	Was a statistically significant difference between groups observed?	Time of assessment	Quality classification
Kelly 2000 [29]	Healthy	Reaction-time to a mental rotation task	Yes (*p* < .05) Statistically significant decrease post-SM compared to control	Unknown	Medium
Dishman 2002 [17]		MEP amplitudes	Yes (*p* < .05) Statistically significant increase from 20 to 120 s. post-SM compared to control	-Immediately after (each 20s during 120 s after SM or control) − 5 min − 10 min	Medium
Dishman 2008 [18]		MEP amplitudes	Yes (*p* < .05) Statistically significant increase at 10 s. post-SM compared to control	Immediately after (each 10s during 100 s after SM or control)	Low
Fryer 2012 [16]		MEP amplitudes	Yes (*p* = .04) Statistically significant decrease post-SM compared to control	Unknown (approximately 10 min after one intervention or the other)	Medium
		MEP latencies	No		
		CSP durations	No		
Ogura 2011 [19]	Symptomatic (mechanical neck pain and shoulder stiffness)	Regional cerebral metabolic rate	Yes (*p* < .001) Statistically significant increase post-SM compared to control in the inferior prefrontal cortex, anterior cingulate cortex, and middle temporal gyrus; and statistically significant decrease post-SM compared to control in the cerebellar vermis and visual association cortex	Between 35 to 55 min	Low
Inami 2017 [8]		Regional cerebral metabolic rate	Yes (*p* < .05) Statistically significant increase post-SM compared to control in the Broca’s area, anterior cingulate cortex, somatosensory association cortex, Wernike’s area, visual association cortex, cerebellar vermis, and visual cortex; and statistically significant decrease post-SM compared to control in the inferior parietal lobule, frontal pole, inferior frontal gyrus, pars triangularis, premotor area/supplementary motor area, primary motor cortex, frontal eye field, dorsolateral prefrontal cortex, angular gyrus, fusiform gyrus, inferior temporal gyrus, and temporal pole.	Between 35 to 65 min	Low
Haavik 2010b [21]	“Subclinical neck/spinal pain”	P14-N18 SEP peak ratio	No	Unclear (said to be within 25 min post-SM or control, possibly 45 min after one intervention or the other)	Medium
		N20-P25 SEP peak ratio	No		
		P22-N30 SEP peak ratio	Yes (*p* = .00005) Statistically significant decrease post-SM compared to control		

Results are reported (i) grouped by type of study subjects (healthy, symptomatic, or with “subclinical neck/spinal pain”), (ii) grouped by type of outcomes, and (iii) consecutively by year of publication

*CSP* Cortical silent period, *MEP* Motor evoked potential, *SEP* Somatosensory evoked potential, *SM* Spinal manipulation

**Table 11:** Results from four studies included in a systematic review on the effect of spinal manipulation on ‘brain function’, comparing spinal manipulation to another physical stimulus

1st Author Year Ref	Type of study subjects	Outcome	Was a statistically significant difference between groups observed?	Time of assessment	Quality classification
Dishman 2008 [18]	Healthy	MEP amplitudes	Yes (*p* < 0.05) Statistically significant increase at 10 s. post-SM compared to control	Immediately after (each 10 s. during 100 s. after SM or control)	Low
Haavik 2010a [26]	“Subclinical neck/spinal pain”	P14-N18 SEP peak ratio	No	Unclear (said to be within 25 min post-SM or control)	Medium
		N20-P25 SEP peak ratio	No		
		P22-N30 SEP peak ratio	Yes (*p* = .003) Statistically significant decrease post-SM compared to control		
Haavik 2016 [24]		MEP amplitudes	Yes (*p* = .01) Statistically significant increase post-SM compared to control	Not-reported	Low
		k (slope of the steepest part of the curve)	No		
		S_50_ (stimulus intensity to obtain a response 50% of the maximum)	No		
Christiansen 2018 [28]		V-wave amplitudes	Yes (*p* < 0.01–0.03) Statistically significant increase at each time point post-SM compared to control	-Immediately after −30 min after − 60 min after	Acceptable

Results are reported (i) grouped by type of study subjects (healthy, symptomatic, or with “subclinical neck/spinal pain”), (ii) grouped by type of outcomes, and (iii) consecutively by year of publication

*MEP* Motor evoked potential, *SEP* Somatosensory evoked potential, *SM* Spinal manipulation

#### Summary of finding in relation to the research questions 1–3

In summary, and in relation to our three first research questions, three studies reported a transient (immediately to about 20 min post-intervention) *effect* on ‘brain function’ of varied types after SM vs. a sham comparator in symptomatic subjects and in “subclinical neck/spinal pain” subjects. However, in these studies SM was compared to sham procedures with unclear credibility, or discovered as such by the study subjects. Also, the experimental findings were untested in relation to clinical benefits. Detailed results are reported in the next section.

#### 1 - Is there an *effect* of SM on ‘brain function’? (*n* = 3)

**Symptomatic subjects (*n*** **= 1)**

One study of ‘acceptable’ methodological quality [9], conducted on subjects suffering from mechanical neck pain, reported an effect on activation in response to noxious stimuli, as measured by fMRI using the blood oxygenation level-dependent (BOLD) signal, after SM vs. a sham comparator. As shown previously, mechanical noxious stimulation resulted in increased activation in several brain areas associated with pain processing. A group comparison was reported, although it was unclear whether it was performed on the pre- post-intervention differences, as it should. This indicated increased activation in the SM group relative to the sham group in the insular cortex, supramarginal gyrus and superior parietal lobe (presumably in sensory association/integration areas). On the other hand, there was increased activation in the sham group relative to the SM group in the cingulate cortex, the supplementary motor area, and the middle temporal gyrus.

**“Subclinical neck/spinal pain” subject (*n*** **= 2)**

Two studies on “subclinical neck/spinal pain” subjects [14, 15], both of ‘medium’ methodological quality, reported an effect of SM vs. a sham comparator. One of these studies found a statistically significant decrease of N30 somatosensory evoked potential peak amplitudes post-SM compared to a sham group [14]. The other one reported a statistically significant decrease of cerebellar inhibition following SM compared to a sham intervention [15].

#### 2 - If there is an *effect*, for how long does it last? (*n* = 3)

**Symptomatic subjects (*n*** **= 1)**

The effect reported by Sparks et al. 2017 [9] (in a study of ‘acceptable’ methodological quality) on symptomatic subjects was immediate, with no effect investigated beyond this time point.

**“Subclinical neck/spinal pain” subject (*n*** **= 2)**

The effect reported by Baarbé et al. 2018 [15] (in a study of ‘medium’ methodological quality) on “subclinical neck pain” subjects was measured after intervention only once, at about 20 min post-intervention. Another potentially relevant study (Lelic et al. 2016) [14], also of ‘medium’ methodological quality, did not report the time of assessment after interventions.

#### 3- If there is an *effect*, is it associated with any clinical benefits? (*n* **= 3)**

**Symptomatic subjects (*n*** **= 1)**

The study by Sparks et al. 2017 [9] on subjects suffering from mechanical neck pain (a study considered to be of ‘acceptable’ methodological quality), in addition to assessing brain activation in response to a noxious stimulus by means of fMRI, assessed pain intensity pre- post-interventions. However, they did not investigate whether there was an association between pain intensity changes and cerebral activity changes, making it impossible to answer this third research question. It is worth noting that the authors investigated whether there was a correlation between subjective ratings of the noxious stimulus intensity and change in activation in the insular cortex, but no such relationship was found.

**“Subclinical neck/spinal pain” subjects (*n*** **= 2)**

None of the two studies on “subclinical neck/spinal pain” subjects [14, 15], both of ‘medium’ methodological quality, included clinical outcomes.

***Other types of controls, specifically ‘inactive control’ (Table*** ***10*** ***) or ‘another physical stimulus (Table*** ***11*** ***), i.e the (‘differences in outcome’ studies (n = 10)***

#### 4 - Is there a *difference* in ‘brain function’ after SM vs. an ‘inactive control’? (*n* = 7)

Seven studies could be used for the fourth research question [8, 16–19, 21, 29], four considered to be of ‘medium’ methodological quality and three of ‘low’ methodological quality (see Table 12). These studies, reporting on three different types of subjects, investigated the potential *changes* on ‘brain function’ in response to SM by using varied outcome measures and/or experimental protocols, which makes comparisons between studies difficult.

**Table 12:** Summary of quality scores and quality classification for 18 articles included in a systematic review on the effect of spinal manipulation on ‘brain function’

Type of study	First author / Year [ref]	Score^a^ (risk of bias and external validity)	Quality classification
Sham studies	Sparks, 2017 [9]	5.5/7 (79%)	acceptable
	Baarbéé, 2018 [15]	3.5/6 (58%)	medium
	Lelic, 2016 [14]	2.5/6 (42%)	medium
Comparison studies	Christiansen, 2018 [28]	5/6 (83%)	acceptable
	Gay, 2014 [22]	5/7 (71%)	acceptable
	Kelly, 2000 [29]	3.5/6 (58%)	medium
	Dishman, 2002 [17] Haavik-Taylor, 2010a [26] Haavik-Taylor, 2010b [21] Fryer, 2012 [16] Niazi, 2015 [27]	2.5/6 (42%)	medium
	Haavik-Taylor, 2007a [20] Haavik-Taylor, 2007b [25] Haavik-Taylor, 2008 [28] Dishman, 2008 [23] Ogura, 2011 [19] HaaviK, 2016 [24] Inami, 2017 [8]	2/6 (33%)	low

^a^The quality score for each study could range from 0 to 6 or 7, depending on their respective study design and the type of study subjects included. Each quality score was then converted on percentage to allow comparisons. Quality classification: ‘low’: 0–33%; ‘medium’: 34–67%; ‘acceptable’: 68%-100

***Summary of findings in relation to the fourth research question***

In these studies, of ‘low’ or ‘medium’ methodological quality, there were, in general, statistically significant *differences in outcome* between SM and the controls but not necessarily in the same direction. When brain areas were compared, differences were found, but again with some conflicting results. Detailed results are reported in the next section.

**Healthy subjects (*n*** **= 4)**

In healthy subjects, an immediate and transient increase of motor-evoked potential amplitudes after lumbar SM was reported in two studies [17, 18], whereas one reported a decrease of motor-evoked potential amplitude after lumbar SM (approximately 10 min after intervention) [16]. The third reported no statistically significant findings for motor-evoked latencies and cortical silent period durations [16]. Two of these studies were considered of ‘medium’ methodological quality [16, 17] and one of ‘low’ methodological quality [18].

A fourth study [29], of ‘medium’ quality, reported a statistically significant greater decrease of reaction-time to a mental reaction task post-SM vs. post-resting without reporting the time of reassessment.

**Symptomatic subjects (*n*** **= 2)**

The two studies conducted on symptomatic subjects were from the same research team and both of ‘low’ methodological quality [8, 19]; the first that was published being considered by its authors as a “proof of concept” study [19], which apparently led to their second experiment [8]. Both reported a statistically significant increase of regional cerebral metabolic rate (glucose uptake) in some brain areas and a statistically significant decrease of glucose uptake in other brain areas, sometimes with conflicting results (see Table 10).

**“Subclinical neck/spinal pain” subjects (*n*** **= 1)**

In a study on “subclinical neck pain subjects” of ‘medium’ methodological quality [21], the authors reported a statistically significant decrease of the P22-N30 somatosensory potential peak ratio post-SM but a statistically significant increase of this ratio post-control intervention. They found no statistically significant *between*-group differences for the other somatosensory evoked potential peak ratios they investigated.

#### 5 - Is there a *difference* in ‘brain function’ after SM vs. ‘another physical stimulus’? (*n* = 4)

Four articles were able to be used in relation to our fifth research question [18, 24, 26, 28]. Two were of ‘low’, one of ‘medium’, and one of ‘acceptable’ methodological quality (see Table 12). Again, as they were conducted on different types of study subjects and/or most often used different outcomes measures, the possibility of making comparisons between studies was limited.

***Summary of findings in relation to the fifth research question***

Some statistically significant *differences in outcome* between SM and the controls were reported but results were mixed, in studies of ‘low’ to ‘acceptable’ methodological quality. Detailed results are reported in the next section.

**Healthy subjects (*n*** **= 1)**

One study of ‘low’ methodological quality [18], conducted on healthy chiropractic students, reported at 10 s post-intervention statistically significant greater motor-evoked potential amplitudes in the SM group vs. a preloading control group.

**“Subclinical neck/spinal pain” subjects (*n*** **= 3)**

One study on “subclinical neck/spinal pain” subjects [26], of ‘medium’ methodological quality, found a statistically significant decrease of the P22-N30 somatosensory potential peak ratio post-SM vs. post-control [26]. There were no statistically significant *between*-group differences for the other somatosensory evoked potential peak ratios investigated.

Haavik et al. 2016 [24], in a study of ‘low’ methodological quality, reported a statistically significant increase in motor-evoked potential amplitudes in the SM group compared to the control group. They did not find any statistically significant *between*-group differences for two other variables they studied.

Christiansen et al. 2018 [28], in a study of ‘acceptable’ methodological quality, conducted on elite taekwondo athletes with “subclinical spinal pain”, found a statistically significant greater V-wave amplitude post-SM vs. post-control at each time point of assessment (immediately, 30, and 60 min after).

## Discussion

### Summary of findings and their interpretation

This systematic review consists of 18 relevant articles. Once classical risk of bias aspects, necessary in this type of experimental design, had been taken into account, most of these articles were considered of ‘low’ or ‘medium’ methodological quality. In addition, their statistical methods and results sections were often difficult to interpret because of unclear and/or unusual descriptions. For methodological reasons, the results of only 13 of these 18 articles were considered for final analysis.

These 13 articles reported on (i) whether SM has an *effect* on ‘brain function’ compared to a sham intervention, and (ii) whether SM *alters* ‘brain function’ in a different way compared to an ‘inactive control’ or ‘another physical stimulus’, and this on different type of study subjects. Based on the studies using a sham intervention as comparator to SM, it seems that SM does have an *effect* on ‘brain function’. As a result, we also studied our two other main objectives, namely (i) how long this *effect* would last, and (ii) whether this *effect* was associated with clinical benefits.

The three studies using a sham intervention as comparator, two of ‘medium’ and one of ‘acceptable’ methodological quality, provided some evidence to support the hypothesis that SM has supra-segmental neurophysiological *effects*. It was thus shown that SM seems to have the potential to transiently alter (i) somatosensory integration of afferent inputs from the upper limb [14], (ii) cerebellar inhibition [15], both on “subclinical neck/spinal pain” subjects and (iii) activation of several brain areas associated with pain processing on acute or subacute mechanical neck pain subjects [9]. However, none reported whether such *effects* were lasting and none investigated whether these were associated with any clinical benefits. Also, according to the authors of these three studies, what these *effects* mean for the brain remains to be established.

The 10 studies not using a sham intervention as comparator, most of which were of ‘low’ or ‘medium’ methodological quality, also reported statistically significant *between*-group *differences* but not necessarily in the same direction and also not systematically for each of the outcomes they studied. Most of these studies reported on healthy or on “subclinical neck/spinal pain” subjects.

In summary, based on both the ‘*effect’* studies and the *‘differences in outcome’* studies, it seems that something does indeed happen within the brain in response to SM. However, what this means remains elusive both in the brain and at a clinical level, and the researchers provide only hypotheses rather than interpretations. In addition, the reported findings have to be interpreted with caution given the general level of methodological quality (‘low’ or ‘medium’) of the included studies.

Showing that SM is reflected by brain activity does not necessarily mean that something ‘positive’ and clinically relevant happens in response to SM. The significance of any putative *effect* on ‘brain function’ must thus be put into perspective by comparing it to *effects* in response to other types of (comparable) physical stimuli or other types of treatment. The question is therefore: Are the findings in relation to ‘brain function’ specific to SM? For various methodological reasons, none of the studies could clearly answer this question. Furthermore, in order to claim brain involvement in the *effects* of SM it should be expected that changes in ‘brain function’ following SM can be shown related to the desired *clinical effects* of SM. However, no information related to any clinical significance of such findings was unearthed in this review.

### Methodological considerations of our own review

Three databases were searched and only one author applied eligibility criteria to the titles. Thus, it is possible that not all relevant articles on the topic were found. Nevertheless, the additional search of reference lists produced only three additional titles. All the following steps of the screening process and of the data extraction were made independently by at least two of the reviewers.

Most of the articles we obtained reported on experimental studies, in which no clinical outcomes were included. Studies of this type are not strictly comparable to ordinary clinical studies using the randomized clinical trial design, for which well-established critical appraisal tools exist. The quality checklist used in the present review was therefore not standard. For example, some usual risk of bias items, such as allocation concealment were not assessed, as they were judged less relevant for non-clinical randomized controlled trials. Regarding allocation concealment, we assumed that it would be difficult to predict which study subjects would react how regarding the outcome variables used in the included studies. Nevertheless, most of the items we selected consisted of accepted items to evaluate risk of bias [31, 32]. These items related to selection, performance, detection, attrition, and analysis risk of bias. Additional methodological concerns specific to the different studies, voiced by the experts, were summarized in a separate column of the quality checklists for the readers who would be interested in more information. These comments can be used as a basis for discussion on how to proceed with future studies of this type.

Because several of the statistical analyses and/or reporting were unclear and/or unusual, we finally resorted to a ‘benefit-of-the-doubt’ approach. Thus, after many discussions and attempts at interpreting some confusing reports, we deviated from our previous criterion, which was to include in the data synthesis only results of the studies that reported clearly differences *between*-groups or *between*-types of interventions. However, such exceptions were noted explicitly in the Results section, either in the text [9] or in Table 9 [14].

Studies were included also when the outcome variables they tested were not necessarily a reflection of ‘brain function’ only, i.e. some would depend on both segmental and supra-segmental changes (e.g. motor evoked potential amplitudes, V-waves, and cortical silent period duration) [12, 34]. This means that results obtained via these outcome variables must be interpreted with caution; a fact that is often admitted by the authors of the reports. On the other hand, being unrestrictive allowed us to cover the literature on the topic more exhaustively.

Many different outcome variables are used in research to measure brain activity, and this was also the case for the articles we included. Their heterogeneity in relation to (i) study subjects (healthy, symptomatic, and “subclinical neck/spinal pain”), (ii) outcome variables (16 different outcome variables for 18 articles), (iii) experimental protocols for each single variable, and (iv) generally rather low methodological quality, makes comparison of studies difficult and one or several meta-analyses impossible.

### Methodological considerations of the included studies

The methodological quality was quite low in relation to well accepted risk of bias items. Admittedly, these types of studies require a lot of knowledge on technical aspects but this must not remove focus from the fundamental methodological requirements of research, namely the necessity to collect and interpret data in an objective manner.

For example, the studies considered to be of ‘low’ and ‘medium’ methodological quality often failed to report using either a credible sham comparator or having been conducted on naïve subjects. As suggested by Fryer and Pearce [16], the blinding or naivety of the study subjects when ‘objective’ outcomes are used could potentially be considered not as important in purely experimental studies. ‘Objective’ here means that study subjects cannot usually willfully or inadvertently influence outcome. However, the *placebo effect* implies complex neurophysiologic responses involving the brain [35]. In our opinion, this makes the use of a sham comparator and the evaluation of its success relevant also for the ‘objective’ outcome measurements used in the included studies.

According to this review, the credibility of the sham comparator used in the three ‘*effect’* studies must be considered uncertain for two and was recognized as such by the participants in the third. Thus it cannot be ruled out that the *effect* of SM on ‘brain function’ was the result of contextual factors, rather than truly caused by the SM, as discussed by Rossettini et al. 2018 [36]. This was acknowledged in one of these reports [9], where the authors noted that changes in cerebral activation in response to noxious stimuli post-SM may reflect subjects’ expectations.

In relation to the ‘*difference in outcome’* studies, the origin of the study subjects was reported in only a few cases. Thus we do not know if they had any preconceived ideas/expectations with respect to the study outcomes. This problem could be compounded if several studies were conducted on the same study subjects.

Another example was that the blinding of the assessor and of the person who analyzed the data was generally poorly reported. Although it is fair to recognize that this reporting may be unusual in some fields of research (e.g. neuroimaging studies), some authors were transparent in relation to this point, which should encourage other researchers also to do the same.

In addition, comments were provided by our experts, suggesting that several of the experimental protocols of these reports lacked some of the standards, specific to such studies. Several comments were also provided from the experts on the statistical analyses, indicating that this was an area of concern, as the statistical analysis is at the heart of the validity of any statistically significant findings.

Neuroimaging studies, which produce ‘visual’ answers, are perhaps easier to interpret for people without specific knowledge in neurological testing. Nevertheless, they present a challenge for formal analysis. For example, quantification of data is difficult. There are many analytic techniques available for these types of studies and there is a lack of consensus with respect to the most appropriate statistical thresholds to be used [37]. Therefore, this type of study needs to be replicated by other independent research teams. Obviously, this is required for any type of research, particularly when one specific research team dominates the area or when there are potential or real conflicts of interest.

### Conceptual concerns

#### In relation to all the studies

The rationale for investigating whether SM acts through modulation of ‘brain function’ was generally not clear in the included studies. Nevertheless, most studies proposed that *changes* observed at the brain level would result (at least partially) from a ‘bottom-up’ mechanism, due to altered afferent inputs in response to SM [8, 9, 14, 15, 17, 18, 20, 21, 23–28]. However, in addition to ‘bottom-up’ effects, SM might change brain activity through ‘top-down’ effects, i.e. through contextual factors. This means that in absence of truly blinded subjects one cannot exclude a ‘top-down’ effect. In addition, a blinded assessor would be required. These are two methodological aspects often lacking in the reports we scrutinized, and therefore ‘top-down’ effects cannot be ruled out to explain some findings.

#### In relation to the studies using “subclinical neck/spinal pain” subjects

Articles, in which Haavik was one of the authors, included “subclinical neck/spinal pain” subjects in their studies [14, 15, 20, 21, 23–28]. However, the definitions of “subclinical neck/spinal pain” were not consistent in the various studies, so this concept remains unclear. In fact, it is uncertain whether these “subclinical neck/spinal pain” study subjects are clearly different from ‘ordinary’ healthy subjects in terms of neurophysiological parameters, such as somatosensory evoked potentials and motor evoked potentials [15, 38]. Most authors of these articles proposed that the *effects* or *changes* they measured in the SM groups reflect *improvement* of ‘brain function’ [14, 15, 20, 21, 23–28]. This, obviously, raises the following question: If these subjects are not different from healthy subjects, what, exactly, would be improved?

Additionally, these studies rest on the assumption that it is possible to detect ‘subluxations’ in people with “subclinical neck/spinal pain”; a concept that remains hypothetical. Overall, it is our considered opinion that some clarifications are needed regarding this “subclinical neck/spinal pain” with ‘subluxations’ concept to ensure appropriate interpretation of the results of these studies.

### Gap between scientific level of evidence and its implementation in clinical practice

FN practitioners use SM as a treatment of ‘brain lesions’ [5] despite a lack of evidence of its clinical effect, as unearthed in this review. One example of how this concept is taught within FN is the seminar in which P Freud, a chiropractor, proposes to show how to ‘adjust the brain’ [39]. Furthermore, this is stated to be based on the latest scientific knowledge on the topic (as shown in Additional file 4).

### Gap between scientific level of evidence and its modification for popularization

Based on this systematic review, we conclude that there is presently no evidence indicating that SM has a beneficial effect on ‘brain function’ or that the diverse findings identified in this review would be in any way indicators of a health benefit in general. It is therefore premature for authors of this type of research to promote clinical benefits. Nevertheless, this can be observed in at least some commercial videos [40] and in an ‘information’ book for patients subtitled “A quest to understand Chiropractic from the inside out” [41]. This information provided to laymen regarding published research on the topic claims clinical effects on ‘brain function’ following SM, contrary to the more careful interpretations in research articles produced by the same researcher [14, 15, 20, 21, 23–28].

In an example of this overreach, Haavik wrote in her book: “Having your spine checked regularly, to ensure your brain is accurately aware of what is going on in and around your body, should be just as common as exercising every day and brushing your teeth. Everyone should have access to chiropractic care right from birth through to the day they pass away. I believe a lot of suffering could be prevented if this was the case.” [41].

Another example is extracted from one of Haavik’s commercial videos [42], which is mainly based on one of her own studies included in the present review [14]. This video starts with the following message: “Chiropractic care really does change brain function!”. After having given a lay interpretation of this study [14], it concludes: “Have you seen your chiropractor lately? You may want to have your brain’s conductor fine-tuned too”. It would be easy to interpret this as a suggestion that the brain is unable to do its job properly but that a chiropractor can improve the situation. However, the section “*Study Considerations”* of that article [14] obviously does not support such claims and the authors of that article point out that it is not clear how long the changes observed in the brain last. They also state that it is not known if the observed changes are, at all, beneficial. There is therefore a gap between the guarded discussion in the peer-reviewed study and the more positive message of the commercial material [42].

## Recommendations

### Recommendations for future research

Further research should be undertaken in this area and we recommend attention to the following:

- The clinical relevance of any brain changes should be investigated using symptomatic study subjects. Also, clinical outcomes should be included and the correlation between these and brain changes should be tested to establish if there is some type of benefits.
- To study the specific effect of SM, proper sham procedures must be adopted and checked for success after the intervention to control for any unspecific effects, including placebo responses.
- Appropriate methodology in relation to randomized controlled trials, with appropriate attention to the potential sources of bias (e.g. blinding of study subjects, assessor, and statistician) should be respected.
- In relation to the technical procedures, standard protocols should be employed to ensure reproducibility of the outcome measures.
- Appropriate statistical methods and thresholds should be used.
- Any conflict of interest should be reported.
- Results should be replicated by independent research teams before their clinical acceptance.

### Recommendations for the chiropractic profession

Presently, the chiropractic profession might wish to consider the potential consequences of encouraging undergraduate- and postgraduate courses on chiropractic approaches relating to the treatment of the brain via the spine. Obviously, this is also true for other health care providers who may be tempted to practice following such concepts in relation to SM. Moreover, statements aimed at the public relating to this phenomenon should be discussed as findings of unknown clinical effect.

## Conclusion

According to the results of the present systematic review, it is premature to promote the use of SM as a treatment to improve ‘brain function’ from a clinical point of view.

Further, studies on this topic should (i) include symptomatic subjects, (ii) relate to the clinical significance by using outcomes relevant to test associations with neurophysiological changes, and (iii) take into consideration basic methodological and technical requirements pertaining to this type of randomized controlled trials.

## Additional files

Additional file 1: Search strategy developed for PubMed for a systematic critical review of the literature on the effect of spinal manipulation on ‘brain function’ (DOCX 126 kb)

Additional file 2: Items selected for the quality checklists and their rationale in relation to a systematic critical review on the effect of spinal manipulation on ‘brain function’ (DOCX 22 kb)

Additional file 3: The various ways used in the scientific literature for testing the hypothesis that spinal manipulation would have an effect on ‘brain function’ (DOCX 83 kb)

Additional file 4: Commercial announcement of a chiropractic seminar entitled “Adjusting the Brain” (PDF 1462 kb)

## Data Availability

The scientific articles scrutinized during the current critical review are available from the corresponding author on reasonable request.

## Abbreviations

BOLD: Blood oxygenation level-dependent
fMRI: Functional magnetic resonance imaging
FN: Functional Neurology
SM: Spinal manipulation
