# Supplementary material for: Unravelling functional neurology: does spinal manipulation have an effect on the brain? - a systematic literature review
Source: Chiropr Man Therap. 2019 Oct 2;27:60. doi: 10.1186/s12998-019-0265-8 (PMC6788096; doi:10.1186/s12998-019-0265-8)
Supplement: Supplementary file 1 — Search strategy developed for PubMed for a systematic critical review of the literature on the effect of spinal manipulation on ‘brain function’ (DOCX 126 kb) [file 12998_2019_265_MOESM1_ESM.docx]

**Additional file 1**: Search strategy developed for PubMed for a systematic critical review of the literature on the effect of spinal manipulation on ‘brain function’

**(“manipulation, spinal”[MH]**OR “spinal manipulation”[TW] OR “spine manipulation”[TW] OR “spinal manipulative therapy”[TW] OR “manipulation”[TI] OR “manipulation”[OT] OR “lumbar manipulation”[TW] OR “cervical manipulation”[TW] OR “thoracic manipulation”[TW] OR “sacroiliac manipulation”[TW] OR “joint manipulation”[TW] OR “peripheral manipulation”[TW] OR **“musculoskeletal manipulations”[MH]** OR “manual therapies”[TW] OR “manual therapy”[TW] OR “manipulation therapy”[TW] OR “manipulation therapies”[TW] OR “manipulative therapy”[TW] OR “manipulative therapies”[TW] OR **“manipulation, orthopedic”[MH]** OR “orthopedic manipulation”[TW] OR **“chiropractic”[MH]** OR **“manipulation, chiropractic”[MH]** OR “chiropractic manipulation”[TW] OR “chiropractic adjustment”[TW] OR **“manipulation, osteopathic”[MH]**OR “osteopathic manipulative treatment”[TW] OR “osteopathic manipulative treatments”[TW] OR “osteopathic manipulation”[TW] OR “osteopathy”[TW] OR “maitland mobilization”[TW] OR “glide mobilization”[TW] OR “HVLA”[TW] OR “high-velocity, low-amplitude spinal manipulation”[TW] OR “high-velocity low-amplitude spinal manipulation”[TW]**) AND** (**“brain”[MH]**OR “encephalon”[TW] OR “encephalons”[TW] OR **“cerebrum”[MH]** OR “cerebral hemispheres”[TW] OR “cerebral hemisphere”[TW] OR **“cerebral cortex”[MH]**OR “cortex, cerebral”[TW] OR **“basal ganglia”[MH]** OR **“cerebellum”[MH]** OR “cerebellums”[TW] OR **“brain stem”[MH]** OR “brainstem”[TW] OR “brainstems”[TW] OR “brain stems”[TW] OR **“thalamus”[MH]** OR **“hypothalamus”[MH] OR** **“transcranial magnetic stimulation”[MH]**OR “magnetic stimulation, transcranial”[TW] OR “stimulation, transcranial magnetic”[TW] OR “transcranial magnetic stimulations”[TW] OR **“magnetoencephalography”[MH]** OR **“electroencephalography”[MH]** OR “EEG”[TW] OR “electroencephalogram”[TW] OR “electroencephalograms”[TW] OR “fMRI”[TW] OR “MRI, functional”[TW] OR “functional MRI”[TW] OR “functional MRIs”[TW] OR **“evoked potentials”[MH]** OR “evoked potential”[TW] OR **“positron-emission tomography”[MH]** OR “positron emission tomography”[TW] OR “PET scan”[TW] OR “PET scans”[TW] OR “scan PET”[TW] OR “scans, PET”[TW] OR “tomography, positron-emission”[TW] OR “radionuclide tomography”[TW] OR **“tomography, emission-computed, single-photon”[MH]** OR “tomography, positron emission”[TW] OR “single-photon emission computerized tomography”[TW] OR “single photon emission computerized tomography”[TW] OR “single-photon emission CT scan”[TW] OR “single photon emission CT scan”[TW] OR “single-photon emission-computed tomography”[TW] OR “single photon emission computed tomography”[TW] OR “SPECT”[TW] OR “tomography, single-photon emission-computed”[TW] OR “single-photon emission computer-assisted tomography”[TW] OR “single photon emission computer-assisted tomography”[TW]**)**
