# Supplementary material for: Unravelling functional neurology: does spinal manipulation have an effect on the brain? - a systematic literature review
Source: Chiropr Man Therap. 2019 Oct 2;27:60. doi: 10.1186/s12998-019-0265-8 (PMC6788096; doi:10.1186/s12998-019-0265-8)
Supplement: Supplementary file 2 — Items selected for the quality checklists and their rationale in relation to a systematic critical review on the effect of spinal manipulation on ‘brain function’ (DOCX 22 kb) [file 12998_2019_265_MOESM2_ESM.docx]

**Additional file 1:** Items selected for the quality checklists and their rationale in relation to a systematic critical review on the effect of spinal manipulation on ‘brain function’

The number of points (pt) given for each item is reported in brackets and the way we assessed them is explained when needed. Items 1-4 and 6-8 relate to risk of bias assessment and item 5 to external validity.

**In relation to study subjects:**

**1 Were study subjects in sham controlled studies reported to be blind?** (Yes / No / Unclear) **If yes / unclear, was the blinding tested for success?** (Yes / No) **If yes, was it successful?** (Yes / No) **(1pt)**

**2 Were study subjects in studies with a control group reported to be naive?** (Yes / No / Unclear) **Was the origin of the subjects reported?** (Yes / No) **If yes, does it allow to exclude any interest?** (Yes / No / Unclear) **(1pt)**

The reason why it is important that subjects are blind or at least naïve to the nature of the experiment is that they may otherwise be influenced by their expectations to treatment outcomes.

We considered that it was important that the origin/source of the subjects was reported in order to check the credibility of their naivety in relation to the outcome of the study.

Because blinding of the subjects is difficult in manual therapies, we expected that the success of the blinding was tested at the end of the study by a questionnaire.

**For studies comparing spinal manipulation (SM) to a sham procedure**:

**One point** was given when the study subjects were reported to be blinded and the success of the blinding of the subjects was confirmed by a questionnaire at the end of the experiment.

**Half a point** was given when the subjects were reported to be blinded but the success of the blinding was not assessed.

**No point** was given when subjects were reported to be blinded but blinding was assessed and reported as unsuccessful for most of the study subjects.

**For studies comparing SM to another control procedure**:

**One point** was given when the subjects were reported as naïve and this was credible, i.e. when study subjects were clearly identified as not being students or practitioners with an interest in SM.

**Half a point** was given (i) when study subjects were reported as naïve but their origin was not reported or did not allow to exclude they had some interest in the outcome of the study, or (ii) when study subjects were not reported as naïve but that their origin indicated that there was probably no interest in the study outcome(s).

**No point** was attributed when subjects were reported as naïve but were identified by the reviewers as potentially having an interest in the outcome of the study (i.e. students or practitioners with an interest with SM).

**3 Were study subjects reported to have been randomly allocated to study groups?** (Yes / No / Unclear) **(1pt)**

The random allocation minimizes risk of selection bias. We only considered if such a procedure was mentioned **(1pt)** or not **(0pt)**, meaning that we did not deal with the appropriateness of the method of randomization. If the study used a crossover design, the order of the interventions should be randomized. We did not investigate concealment because we did not think it is possible for examiners to ‘pick’ study subjects who would react in one way or the other to this type of experimental study.

**4 Were study groups comparable in relation to symptoms when studying symptomatic subjects** (duration and pain intensity)**?** (Yes / No) **(1pt)**

This should allow for relevant comparisons between groups in studies including symptomatic subjects. Subjects categorized as in “subclinical neck/spinal pain” were considered as comparable in relation to symptoms, meaning that this item was not applicable. This item was also considered irrelevant for studies conducted in a cross-over design.

**In relation to the experiment (including the assessment):**

**5 Were the intervention and control(s) well described** (at least where and how)**?** (Yes / No) **(1pt** _ 0.5pt for intervention and 0.5pt for control(s)**)**

The SM should be well described, i.e. the area(s) of the spine where the SM was provided and the type of SM (manually or mechanically/instrumentally assisted) should be reported. Sufficient details in relation to the tested intervention should allow for replication of the study. In a similar manner, also controls should be described.

**6 Was the assessor reported to be blind to group allocation?** (Yes / No) **(1pt)**

When not blinded, the assessor may be influenced by his/her wish to obtain better or worse results in the intervention group compared to the sham/control group (expectation bias). Results could thus be transcribed or interpreted in a biased manner. In addition, the behavior of the assessor could have a placebo or nocebo influence on the study subjects.

**In relation to data analysis and data reporting:**

**7 Were losses and exclusions of study subjects reported or obvious in result section** (including tables or graphs)**?** (Yes / No / Unclear) **(1pt)**

Losses and exclusions should be reported to make it possible to appreciate to which extent any losses could affect the reported results.

**8 Was the person who statistically analyzed the data reported to be blind to group allocation?** (Yes / No) **(1pt)**

The person who analyzed the data has the possibility to decide on the removal of outliers, reorganization of data, and choice of statistical methods, for which reason this person should be blind to group allocation.

Some comments by the technical experts on the methodology and/or technical aspects of the included studies were provided in the last column of some of the quality checklists but not used to assess the quality of studies (see Tables 2a-e, col.10). As we did not have access to experts on all the outcomes used in the included studies, comments related to methodology and/or technical aspects could not always be provided (as mentioned in Tables 2b, 2c and 2e, col.10).
