# Supplementary material for: Unravelling functional neurology: does spinal manipulation have an effect on the brain? - a systematic literature review
Source: Chiropr Man Therap. 2019 Oct 2;27:60. doi: 10.1186/s12998-019-0265-8 (PMC6788096; doi:10.1186/s12998-019-0265-8)
Supplement: Supplementary file 3 — The various ways used in the scientific literature for testing the hypothesis that spinal manipulation would have an effect on ‘brain function’ (DOCX 83 kb) [file 12998_2019_265_MOESM3_ESM.docx]

**Additional file 3**: The various ways used in the scientific literature for testing the hypothesis that spinal manipulation would have an effect on ‘brain function’

**Single-pulse** **transcranial magnetic stimulation** (TMS) of the motor cortex may be used to assess excitability along the corticospinal tract (i.e. upper and lower motor neurons) by recording **(i)** *motor-evoked potentials* (MEP) of various muscles, at rest or during slight contraction [1, 2]. MEPs are the motor responses recorded in a muscle following the magnetic stimulation of the area of the motor cortex in charge of its control, stimulation that is adjusted in intensity to produce action potentials along the corticospinal tract. These are assessed using electromyography. Six studies included in the present review used MEP amplitude and/or latency as primary outcome [3-8].

During slight contraction, **(ii)** the *cortical silent period* (CSP), which depends on both spinal and cortical mechanisms [1, 2], can also be recorded with single-pulse TMS. This is a period, just following the occurrence of a motor-evoked potential, where the electromyographic activity is inhibited, partly through a suppression of corticospinal output at the cortical level. Thus, although its first part is attributed to spinal mechanisms, the CSP appears to be considered primarily as a measure of motor inhibition from cortical origin [1, 2]. Three of the selected studies used CSP duration as a primary outcome [4, 5, 7].

**Paired-pulse transcranial magnetic stimulation** of the motor cortex allows to assess some intra-cortical inhibition process such as **(iii)** *short interval intra-cortical inhibition* (SICI) and **(iv)** *long interval intra-cortical inhibition* (LICI); it also allows to assess intra-cortical facilitation process such as **(iv)** *short interval intra-cortical facilitation* (SICF) [2, 9]. Paired-pulse TMS involves two successive stimuli, a *conditioning stimulus* followed by a *test stimulus*, both directed to the same area of the motor cortex, provided at predetermined intensities and intervals, depending on which process would be measured [2]. Only one of the included study used these types of outcome measures [5].

Also, a specific paired-pulse paradigm allows to assess the functional connectivity between the cerebellum (where the *conditioning stimulus* is applied) and the motor cortex (where the *test stimulus* is applied), by assessing the **(v)** cerebellar inhibition (CBI) [10]. All these neurophysiological measures, i.e. SICI, LICI, SICF and CBI, are obtained by recording motor-evoked potentials that are compared to those produced by single-pulse TMS (as such described above) [2, 9, 10]. One of the included studies used CBI as primary outcome [11].

The cortical efferent outputs directed to lower motor neurons may also be investigated by measuring the **(vi)** *volitional wave response*, usually called *V-wave*, a reflex response generated by a muscle which is evoked by a **suprathreshold electrical nerve stimulation** delivered during its voluntary contraction [12, 13]. The *V-wave* is described as potentially altered by both segmental and supra-segmental mechanisms but seems to be considered by some authors as primarily reflecting supra-segmental changes [12, 14, 15], especially when it is used in addition to the H-reflex, a reflex response also evoked by electrical nerve stimulation, which may serve as a control for potential segmental changes [13-15]. Two selected studies used V-wave and H-reflex in attempts to distinguish between cortical and spinal mediated changes in response to spinal manipulation (SM) [16, 17].

Beside cortically mediated motor functions, the ability of the brain to process and to integrate somatosensory peripheral afferent inputs was also investigated after SM in four of the included studies [18-21]. Cortical somatosensory processing and integration of peripheral inputs can be explored by recording **(vii)** cortical *somatosensory evoked potentials* (SEP) following **peripheral nerve stimulation**. A SEP is an electrical potential, usually elicited by electrical stimulation of a peripheral nerve, for example the median nerve, and is recorded by surface electrodes placed along the lemniscal pathways (the pathway of the ascending signal), from the periphery to the cortex [22-24]. Thus, several peripheral, spinal, sub-cortical and cortical SEPs may be recorded following stimulation, each having more or less well identified neural generator(s) [22-24]. This was studied in two of the included studies [18, 21].

Still by recording cortical SEPs, but using a **dual peripheral nerve stimulation somatosensory evoked potential ratio technique**, the ability of the somatosensory cortex to integrate dual sensory inputs from two adjacent body parts, for example coming from the ulnar and from the median nerves, may be investigated [25]. This process refers to **(viii)** cortical *surrounding inhibition* or *reciprocal sensory inhibition* and is described as reflecting the capacity of the somatosensory system, including cortical areas, to enhance the contrast between two stimuli, allowing the information to be perceived and processed separately [25]. This SEP ratio technique was used in two of the included studies [19, 20].

Cognitive function has also been of interest in one of the included study with the use of a **mental rotation task** [26]. This is a cognitive task, during which two stimuli are presented with varying angular disparity and **(ix)** *reaction time* is measured*.* This would be the time between stimuli presentation and the decision of the subject, whether these two stimuli do or do not match when mentally aligned [27]. The *reaction time* is described as the sum of times needed to accomplish four steps that lead to subject decision: 1) stimulus identification, 2) mental rotation of the stimuli, 3) comparison of the stimuli once mentally aligned by rotation and response selection, i.e. do they match or not, and 4) execution or motor response. This type of task is thus used to assess the ability of the subject to mentally rotate two- or three-dimensional stimuli [27, 28].

Functional neuroimaging techniques are other tools to investigated potential alterations in brain function after intervention. **Positron emission tomography** (**PET) combined with the radioactive tracer Fludeoxyglucose (18F)** measures **(x)** *regional cerebral metabolic rates for glucose* as an indirect measure of neuronal activity [29, 30] . When changes of regional metabolic rates for glucose are investigated, the PET-scan makes it possible to assess the cerebral areas that are activated versus those that are deactivated, respectively areas that present an increase versus those that present a decrease in glucose consumption. This imaging results from the emissions produced by the radioactive molecules injected which are detected by the PET-scanner [29, 30]. Two of the included studies used regional cerebral metabolic rate as primary outcome [31, 32].

Finally, two studies [33, 34] chose outcomes assessed by **functional magnetic resonance imaging (fMRI)**. fMRI is based on the same technology as magnetic resonance imaging and aims to detect hemodynamic changes that occur with changes in neural activity in the brain. Several fMRI approaches exist, the most common one being the **(xi)** *blood-oxygen-level-dependent (BOLD) contrast.* Activation studies typically measure BOLD signal changes in response to a stimulus (used by Sparks et al. 2017 [34]). When investigating **(xii)** *functional connectivity*, the BOLD signal across different brain regions is interrogated for temporal coherence (correlation in time) (used by Gay et al. 2014 [33]). Thus, potential changes in the simultaneous activation of different brain areas is investigated through this fMRI approach in order to explore the neurophysiological mechanisms which may underlie a tested intervention.

**LIST OF ABBREVIATIONS**

BOLD: blood-oxygen-level-dependent; CBI: cerebellar inhibition; CSP: cortical silent period; fMRI: functional magnetic resonance imaging; LICI: long interval intra-cortical inhibition; MEP: motor-evoked potential; PET: positron emission tomography; SEP: somatosensory-evoked potentials SICF: short interval facilitation; SICI: short interval intra-cortical inhibition; SM; spinal manipulation; TMS: transcranial magnetic stimulation.

**REFERENCES**

1. Groppa S, Oliviero A, Eisen A, Quartarone A, Cohen LG, Mall V, Kaelin-Lang A, Mima T, Rossi S, Thickbroom GW *et al*: **A practical guide to diagnostic transcranial magnetic stimulation: report of an IFCN committee**. *Clin Neurophysiol* 2012, **123**(5):858-882.

2. Rossini PM, Burke D, Chen R, Cohen LG, Daskalakis Z, Di Iorio R, Di Lazzaro V, Ferreri F, Fitzgerald PB, George MS *et al*: **Non-invasive electrical and magnetic stimulation of the brain, spinal cord, roots and peripheral nerves: Basic principles and procedures for routine clinical and research application. An updated report from an I.F.C.N. Committee**. *Clin Neurophysiol* 2015, **126**(6):1071-1107.

3. Dishman JD, Ball KA, Burke J: **First Prize: Central motor excitability changes after spinal manipulation: a transcranial magnetic stimulation study**. *J Manipulative Physiol Ther* 2002, **25**(1):1-9.

4. Haavik-Taylor H, Murphy B: **Transient modulation of intracortical inhibition following spinal manipulation**. *Chiropr J Aust* 2007, **37**:106-116.

5. Taylor HH, Murphy B: **Altered sensorimotor integration with cervical spine manipulation**. *J Manipulative Physiol Ther* 2008, **31**(2):115-126.

6. Dishman JD, Greco DS, Burke JR: **Motor-evoked potentials recorded from lumbar erector spinae muscles: a study of corticospinal excitability changes associated with spinal manipulation**. *J Manipulative Physiol Ther* 2008, **31**(4):258-270.

7. Fryer G, Pearce AJ: **The effect of lumbosacral manipulation on corticospinal and spinal reflex excitability on asymptomatic participants**. *J Manipulative Physiol Ther* 2012, **35**(2):86-93.

8. Haavik H, Niazi IK, Jochumsen M, Sherwin D, Flavel S, Turker KS: **Impact of Spinal Manipulation on Cortical Drive to Upper and Lower Limb Muscles**. *Brain Sci* 2016, **7**(1).

9. Valero-Cabre A, Pascual-Leone A, Coubard OA: **[Transcranial magnetic stimulation (TMS) in basic and clinical neuroscience research]**. *Rev Neurol (Paris)* 2011, **167**(4):291-316.

10. Baarbe J, Yielder P, Daligadu J, Behbahani H, Haavik H, Murphy B: **A novel protocol to investigate motor training-induced plasticity and sensorimotor integration in the cerebellum and motor cortex**. *J Neurophysiol* 2014, **111**(4):715-721.

11. Baarbe JK, Yielder P, Haavik H, Holmes MWR, Murphy BA: **Subclinical recurrent neck pain and its treatment impacts motor training-induced plasticity of the cerebellum and motor cortex**. *PLoS One* 2018, **13**(2):e0193413.

12. Grospretre S, Martin A: **Conditioning effect of transcranial magnetic stimulation evoking motor-evoked potential on V-wave response**. *Physiol Rep* 2014, **2**(12).

13. McNeil CJ, Butler JE, Taylor JL, Gandevia SC: **Testing the excitability of human motoneurons**. *Front Hum Neurosci* 2013, **7**:152.

14. Aagaard P, Simonsen EB, Andersen JL, Magnusson P, Dyhre-Poulsen P: **Neural adaptation to resistance training: changes in evoked V-wave and H-reflex responses**. *J Appl Physiol (1985)* 2002, **92**(6):2309-2318.

15. Vila-Cha C, Falla D, Correia MV, Farina D: **Changes in H reflex and V wave following short-term endurance and strength training**. *J Appl Physiol (1985)* 2012, **112**(1):54-63.

16. Niazi IK, Turker KS, Flavel S, Kinget M, Duehr J, Haavik H: **Changes in H-reflex and V-waves following spinal manipulation**. *Exp Brain Res* 2015, **233**(4):1165-1173.

17. Christiansen TL, Niazi IK, Holt K, Nedergaard RW, Duehr J, Allen K, Marshall P, Turker KS, Hartvigsen J, Haavik H: **The effects of a single session of spinal manipulation on strength and cortical drive in athletes**. *Eur J Appl Physiol* 2018, **118**(4):737-749.

18. Haavik-Taylor H, Murphy B: **Cervical spine manipulation alters sensorimotor integration: a somatosensory evoked potential study**. *Clin Neurophysiol* 2007, **118**(2):391-402.

19. Taylor HH, Murphy B: **Altered central integration of dual somatosensory input after cervical spine manipulation**. *J Manipulative Physiol Ther* 2010, **33**(3):178-188.

20. Haavik Taylor H, Murphy BA: **Altered cortical integration of dual somatosensory input following the cessation of a 20 min period of repetitive muscle activity**. *Exp Brain Res* 2007, **178**(4):488-498.

21. Lelic D, Niazi IK, Holt K, Jochumsen M, Dremstrup K, Yielder P, Murphy B, Drewes AM, Haavik H: **Manipulation of Dysfunctional Spinal Joints Affects Sensorimotor Integration in the Prefrontal Cortex: A Brain Source Localization Study**. *Neural Plast* 2016, **2016**:3704964.

22. Cruccu G, Aminoff MJ, Curio G, Guerit JM, Kakigi R, Mauguiere F, Rossini PM, Treede RD, Garcia-Larrea L: **Recommendations for the clinical use of somatosensory-evoked potentials**. *Clin Neurophysiol* 2008, **119**(8):1705-1719.

23. Passmore SR, Murphy B, Lee TD: **The origin, and application of somatosensory evoked potentials as a neurophysiological technique to investigate neuroplasticity**. *J Can Chiropr Assoc* 2014, **58**(2):170-183.

24. Macerollo A, Brown MJN, Kilner JM, Chen R: **Neurophysiological Changes Measured Using Somatosensory Evoked Potentials**. *Trends Neurosci* 2018, **41**(5):294-310.

25. Tinazzi M, Priori A, Bertolasi L, Frasson E, Mauguiere F, Fiaschi A: **Abnormal central integration of a dual somatosensory input in dystonia. Evidence for sensory overflow**. *Brain* 2000, **123 ( Pt 1)**:42-50.

26. Kelly DD, Murphy BA, Backhouse DP: **Use of a mental rotation reaction-time paradigm to measure the effects of upper cervical adjustments on cortical processing: a pilot study**. *J Manipulative Physiol Ther* 2000, **23**(4):246-251.

27. Shepard RN, Metzler J: **Mental rotation of three-dimensional objects**. *Science* 1971, **171**(3972):701-703.

28. Jansen P, Schmelter A, Quaiser-Pohl C, Neuburger S, Heil M: **Mental rotation performance in primary school age children: Are differences in chronometric tests?** *Cognitive Development* 2013, **28**(1):51-62.

29. Magistretti PJ, Pellerin L: **Cellular mechanisms of brain energy metabolism and their relevance to functional brain imaging**. *Philos Trans R Soc Lond B Biol Sci* 1999, **354**(1387):1155-1163.

30. Tashiro M, Itoh M, Fujimoto T, Masud MM, Watanuki S, Yanai K: **Application of positron emission tomography to neuroimaging in sports sciences**. *Methods* 2008, **45**(4):300-306.

31. Ogura T, Tashiro M, Masud M, Watanuki S, Shibuya K, Yamaguchi K, Itoh M, Fukuda H, Yanai K: **Cerebral metabolic changes in men after chiropractic spinal manipulation for neck pain**. *Altern Ther Health Med* 2011, **17**(6):12-17.

32. Inami A, Ogura T, Watanuki S, Masud MM, Shibuya K, Miyake M, Matsuda R, Hiraoka K, Itoh M, Fuhr AW *et al*: **Glucose Metabolic Changes in the Brain and Muscles of Patients with Nonspecific Neck Pain Treated by Spinal Manipulation Therapy: A [(18)F]FDG PET Study**. *Evid Based Complement Alternat Med* 2017, **2017**:4345703.

33. Gay CW, Robinson ME, George SZ, Perlstein WM, Bishop MD: **Immediate changes after manual therapy in resting-state functional connectivity as measured by functional magnetic resonance imaging in participants with induced low back pain**. *J Manipulative Physiol Ther* 2014, **37**(9):614-627.

34. Sparks CL, Liu WC, Cleland JA, Kelly JP, Dyer SJ, Szetela KM, Elliott JM: **Functional Magnetic Resonance Imaging of Cerebral Hemodynamic Responses to Pain Following Thoracic Thrust Manipulation in Individuals With Neck Pain: A Randomized Trial**. *J Manipulative Physiol Ther* 2017, **40**(9):625-634.
