# Supplementary material for: Unravelling functional neurology: does spinal manipulation have an effect on the brain? - a systematic literature review
Source: Chiropr Man Therap. 2019 Oct 2;27:60. doi: 10.1186/s12998-019-0265-8 (PMC6788096; doi:10.1186/s12998-019-0265-8)
Supplement: Supplementary file 4 — Commercial announcement of a chiropractic seminar entitled “Adjusting the Brain” (PDF 1462 kb) [file 12998_2019_265_MOESM4_ESM.pdf]

ADJUSTING THE BRAIN

LA SCIENCE, LA PHILOSOPHIE ET L'ART DE LA NEUROLOGIE CHIROPRATIQUE

-Apprenez la recherche qui appuie la chiropratique
-Appliquez la neuroscience dans votre pratique
-Obtenez des résultats supérieurs pour vos patients

ADJUSTING THE BRAIN

TROIS-RIVIÈRES, QUÉBEC (en Français)

- 8 avril 2018
- Accrédité par le National University of Health Sciences

(NUHS) (organisme admissible pour l'OCQ) pour 7 heures de formation continue.

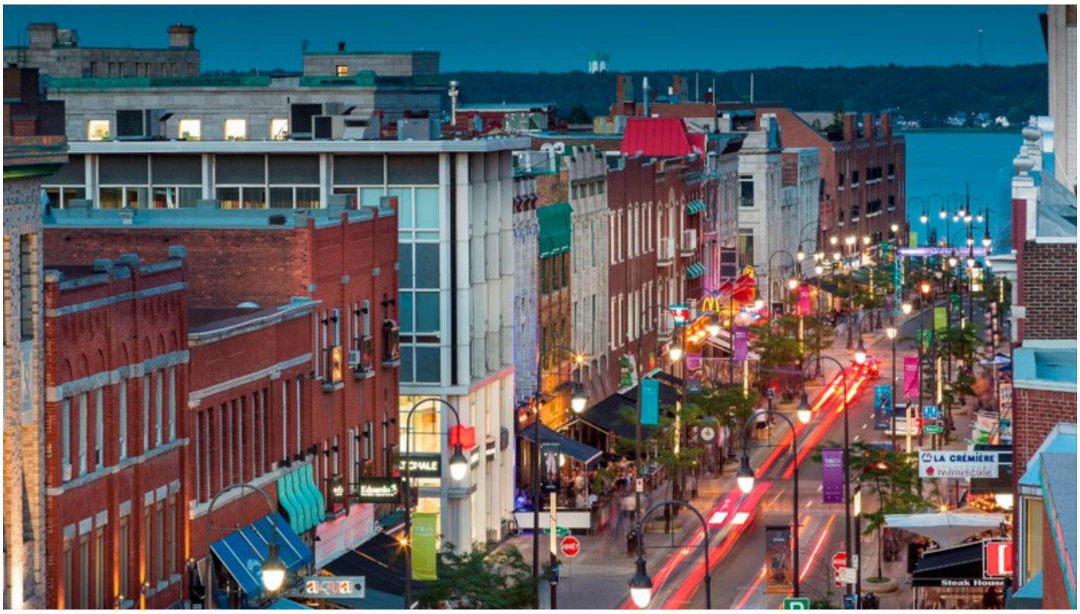

Les recherches en neuroscience nous permettent d’avoir davantage confiance dans les concepts fondamentaux de la chiropratique, tout en obtenant des résultats supérieurs pour nos patients.

Ce séminaire, un cours "Neurologie Chiropratique 101", portera sur les concepts et les applications les plus importantes pour le chiropraticien…

La SCIENCE de la Neurologie Chiropratique :

- Les meilleurs tests neurologiques à effectuer en pratique chiropratique
- La neuroscience de la subluxation
- Les dernières recherches sur les effets de l'ajustement sur le cerveau
- La voie neurologique que tout chiropraticien doit connaître
- Comment Harvey a-t-il récupéré son ouïe?

La PHILOSOPHIE de la Neurologie Chiropratique :

- Est-ce que la subluxation existe vraiment?
- Examinons le cerveau du chiropraticien!
- Un modèle pour une plus grande harmonie en chiropratique (l’unité ne veut pas dire l’uniformité)
- Quel est l'avenir de notre profession?

L'ART de la Neurologie Chiropratique :

- Comment effectuer un examen neurologique significatif pour le chiropraticien
- Quels sont les meilleurs exercices neurologiques à prescrire aux patients?
- Comment modifier les techniques que vous utilisez déjà (chiropratiques et de tissus mous) pour créer un impact neurologique plus profond?
- Comment effectuer des ajustements chiropratiques de la colonne et des extrémités basés sur les principes de la neurologie?
- Des applications pour TOUS vos patients: lombalgies, problèmes de cou, problèmes des extrémités, mauvaise posture, commotions cérébrales, problèmes d'équilibre, TDAH, etc.

Ceci sera un événement à ne pas manquer...

informatif, pratique et inspirant*!*

Cliquez ici pour vous inscrire au séminaire

EMPLACEMENT :

GOUVERNEUR
HÔTELS

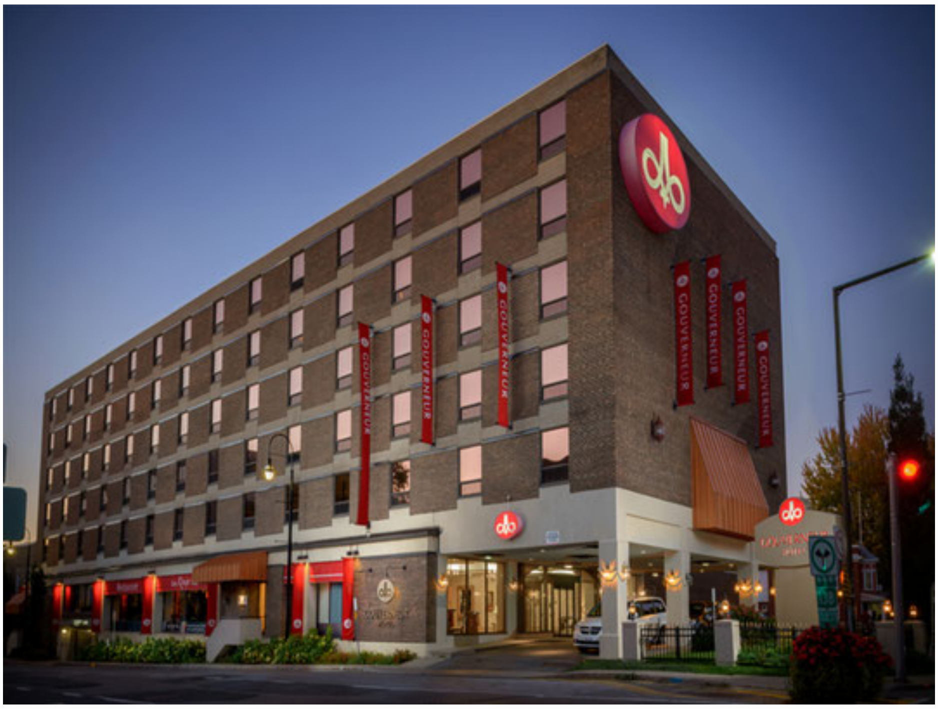

Hôtel Gouverneur Trois-Rivières

975, rue Hart
Trois-Rivières, Québec G9A 4S3

Tél: 888-988-6934

Enregistrement: 8h00 à 8h45

Séminaire: 9h00 à 17h00 (avec pause dîner)

ADJUSTING
THE BRAIN

SÉMINAIRES À VENIR :

TROIS-RIVIERES, QC - 27 janvier 2018 - COMPLET
(Association québécoise de chiropratique pédiatrique et périnatale)

- "Réflexes primitifs et posturaux : Évaluation clinique, applications pratiques et prescription d'exercices chez l'enfant de 0 à 5 ans"
- Contact: Dre Ève Laferrière (info@chirofamille.ca)

TROIS-RIVIERES, QC - 3 février 2018
(Horizons Chiropratique)

- "La neurologie de la communication et le pouvoir de la connection"
- Contact: horizonschiropratique@gmail.com

PARIS, FRANCE (IFEC) - 10 mars 2018 - COMPLET

- “L’application de la neurologie chiropratique, de l’examen au traitement”
- Enregistrement: 8h15 à 8h45
- Séminaire: 9h00 à 17h00 (avec pause déjeuner)
- Contact: info@adjustingthebrainfrancais.com

TROIS-RIVIERES, QC - 8 avril 2018

- "Neurologie Chiropratique 101: La science, la philosophie, et l'art de la neurologie chiropratique"
- Contact: info@adjustingthebrain.com

LAURENTIDES, QC - 28 avril 2018
(ACQ 3L)

- “L’application de la neurologie chiropratique, de l’examen au traitement” (4 heures)
- Contact: Dr Sébastien Fortier (sebfortier@hotmail.com)

DENVER, CO - 21 juillet 2018

- Chiropractic Neurology 101: The Science, Philosophy, and Art of Chiropractic Neurology"
- Contact: info@adjustingthebrain.com

LONGUEUIL, QUÉBEC - 8 septembre 2018
(Symposium Dystonie-Partage pour personnes atteintes de la dystonie)

- La neurologie chiropratique et la dystonie
- Contact: Chantale Boivin (cboivin99@videotron.ca)

SAGUENAY-LAC-ST-JEAN, QC - 20 octobre 2018 (à confirmer)

- "La neurologie chiropratique" (à confirmer)
- Contact: annie-clauderoy.dc@hotmail.com

CLICK HERE FOR ENGLISH SITE

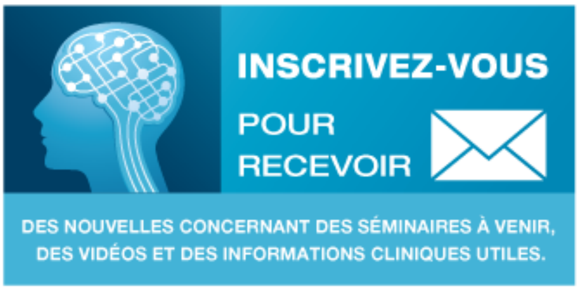

* requis

Titre *

Dr(e)

Prénom *

Nom *

Ville, état/province/région *

Pays *

Email *
